# Unraveling proteomic signatures and neuroinflammatory networks in a CCI rat model of early sciatica: insights for neuropathic pain mechanisms

**DOI:** 10.3389/fnmol.2025.1674151

**Published:** 2025-12-05

**Authors:** Xingjuan Li, Xiaojie Wang, Jinhui Song, Bin Jiang, Yaqin Wen, Yang Wang, Bo Liu, Xiao Zheng

**Affiliations:** 1 School of Bioengineering, Beijing Polytechnic University, Beijing, China; 2 Britton Chance Center for Biomedical Photonics at Wuhan National Laboratory for Optoelectronics-Hubei Bioinformatics & Molecular Imaging Key Laboratory, Department of Biomedical Engineering, College of Life Science and Technology, Huazhong University of Science and Technology, Wuhan, China; 3 Department of Acupuncture and Massage, National Center of Gerontology, Institute of Geriatric Medicine, Chinese Academy of Medical Science, Beijing Hospital, Beijing, China

**Keywords:** sciatica, inflammatory cytokines, neuroinflammation, neuropathic pain, neurotrophic-related proteins

## Abstract

**Introduction:**

Sciatica is a prevalent and highly debilitating condition that is clinically characterized by pain radiating along the distribution of the sciatic nerve. Despite its common occurrence, the progression of early sciatica remains not yet fully elucidated. The aim of this study is to explore the potential molecular mechanism underlying early-stage sciatica progression.

**Methods:**

A total of 20 rats were collected, with 9 in the control group and 11 rats in the chronic constriction injury (CCI) model group. The sciatic nerve tissues of rats were collected at three time points 1, 3, and 7 days post surgery. Protein microarray was used to detect the expression levels of 27 cytokines in sciatic nerve tissues at different times. Gene ontology (GO) and Kyoto Encyclopedia of Genes and Genomes (KEGG) were used for functional and pathway analysis of the differentially expressed proteins (DEPs). ELISA was used to detect the levels of chemokine CINC-2 and neurotrophic growth factors (CNTF).

**Results:**

A total of 11 proteins showed significant differential expression between the CCI and control groups at all three time points (days 1, 3, and 7) after sciatic nerve injury. Specifically, Cytokine-Induced Neutrophil Chemoattractant-2 (CINC-2), Cytokine-Induced Neutrophil Chemoattractant-3 (CINC-3), Lipopolysaccharide-Induced CXC chemokine (LIX), Lymphocyte-Selectin (L-Selectin), Platelet-Derived Growth Factor-AA (PDGF-AA), Interleukin-1 alpha (IL-1α), Interleukin-6 (IL-6), Tissue Inhibitor of Metalloproteinase-1 (TIMP-1), and beta-Nerve Growth Factor (*β*-NGF) were significantly upregulated (*p* < 0.05), whereas the neurotrophic-related protein CNTF was significantly downregulated (*p* < 0.05). KEGG pathway analysis revealed that these DEPs were primarily enriched in key inflammatory signaling pathways, including the JAK–STAT, Cytokine-cytokine receptor interaction, Chemokine, Tumor Necrosis Factor (TNF), NOD-like receptor, and NF-kappa B signaling pathways. GO analysis indicated their involvement in biological processes such as immune response and cellular chemotaxis. Protein function analysis further confirmed the close correlation of these DEPs with cellular recognition and neuroinflammation. Additionally, ELISA validation showed that the key protein CINC-2 was upregulated and CNTF was significantly downregulated in the early CCI group.

**Discussion:**

The progression of early sciatic is closely associated with neuroinflammation triggered by the overexpression of inflammatory factors and nerve dysfunction mediated by neurotrophic-related proteins.

## Introduction

The sciatic nerve is made up of nerve roots from L4 to S3. Different lumbar spine movements, such as bending, flexing and twisting, can exacerbate sciatic discomfort (Davis et al., 2025). Sciatica refers to pain in the distribution of the sciatic nerve caused by lesions of the sciatic nerve itself or the spinal nerve roots (Pinto et al., 2017; Stafford et al., 2007). While typically associated with lumbar disc herniation, up to 30% of cases present without clear structural compression, suggesting important neuroinflammatory mechanisms. The early phase of sciatica (first 7 days) is particularly clinically significant as this window represents a critical period for intervention to prevent chronic neuropathic pain. Sciatica is a relatively common but debilitating condition, with a lifetime incidence rate ranging from 13% to 40%. Correspondingly, the annual incidence of sciatica is between 1% and 5% (Stafford et al., 2007; Zhang et al., 2022a). Intense and long-lasting pain is the norm, and the prognosis is worse than other low back pains. It can disable patients, causing extreme distress, and may end their normal work and social life. It is a pressing clinical disease (Pinto et al., 2017). The molecular mechanism of sciatica is still unclear. Current treatments, including NSAIDs (Rasmussen-Barr et al., 2017), hormones (Ter Meulen et al., 2016), and analgesic/sedative drugs (Sukmawan et al., 2021), often have unsatisfactory efficacy and remarkable adverse reactions. It is urgent to study its molecular mechanism.

Sciatica is most commonly caused by degenerative spinal diseases such as stenosis and disc herniation (Chu and Trager, 2022). Clinical findings show that removing herniated disc material or other causes that lead to nerve root compression does not always relieve pain (Garfin et al., 1991; Rydevik, 1994). Spinal nerve compression causes functional loss, not pain (Mulleman et al., 2006). Analysis of 77 intervertebral disc homogenates removed from patients with nerve root pain showed that the expression levels of inflammatory mediators IL-1α, IL-1β, IL-6, and TNF-α were relatively high. These inflammatory mediators mainly originate from “pathological” intervertebral disc cells and invading macrophages (Takahashi et al., 1996). Inflammatory mediators induce matrix metalloproteinases (MMPs), degrade extracellular matrix (ECM), and aggravate nerve root edema and inflammatory infiltration (Li et al., 2023). This demonstrates the crucial role of inflammatory mediators in the development of sciatica (Khan et al., 2017; Wuertz and Haglund, 2013). Cytokines, chemokines and inflammatory cells are key inflammatory mediators (Denes et al., 2010). The pathophysiology of sciatica involves multiple cellular pathways. Common cellular events include oxidative and nitrosative stress, causing mitochondrial dysfunction, aggravating axonal demyelination (Ullah et al., 2021), and activating chemokine systems (e.g., CCL2) and inflammatory cytokines like TNF, IL-1, and IL-6.

To better understand the progression mechanisms of early-stage sciatica, we used protein chips to detect differentially expressed proteins in the CCI and control groups at 1, 3, and 7 days after nerve injury. We identified key differentially expressed inflammatory and neurotrophic-related proteins between the two groups and elucidated the potential mechanisms underlying early-stage sciatica through pathway enrichment analysis and ELISA validation. This study provides novel insights into the molecular mechanisms of early-stage sciatic progression and suggests a potential target for future therapy.

## Materials and methods

### Animal samples

Twenty Sprague–Dawley specific pathogen free rats, aged 6 weeks, weighing 200–220 g, were purchased from Beijing Vital River Laboratory Animal Technology Co., Ltd., China. These rats were housed under standard conditions (25 ± 1 °C, 12 h light/dark cycle, ad libitum access to food and water). The Institutional Animal Care and Use Committee of Beijing Vital River Laboratory Animal Technology Co., Ltd. approved the experimental procedures (approval No. 2023072601).

### Construction of CCI rat model

Eighteen rats were randomly divided into control group (*n* = 9) and experimental group (CCI group, *n* = 11). The chronic constriction injury was constructed according to the method of Bennett et al. (2003). The rats were anesthetized using a standard anesthesia machine (R520IP, RWD Life Science, Shenzhen, china). specifically, 4%–5% isoflurane was employed for induction, and 2.5%–3.0% isoflurane was utilized for maintenance of anesthesia. Following successful anesthesia, the rats were fixed in prone position on the rat board, dehairing, disinfection, incising the skin. On the right hind limb, approximately 1 cm below the femur and parallel to the femur, the subcutaneous tissues and muscles were bluntly separated to expose the sciatic nerve. The sciatic nerve was freed along a length of approximately 9 mm, and 4 loose ligatures were applied to the sciatic nerve using 4.0 chromic gut suture, spaced approximately 1 mm apart, ensuring a small twitch in the operated hind limb. The incisions were irrigated with 0.9% sodium chloride solution, and the ligatures were sequentially sutured. In the control group, the sciatic nerve was exposed but not ligated, and other procedures were the same as described. After recovery, the rats were housed in individual cages. At 1, 3, and 7 days post sciatic nerve injury, the rats were administered a humane euthanasia procedure using CO_2_. The CO_2_ flow rate for euthanasia was 30–70% of the cage volume per minute. Subsequently, a single sciatic nerve sample was harvested from each rat for subsequent experimental analysis. A segment of the nerve tissue was extracted from the sciatic nerve, and any residual chromic gut was removed in model groups. The nerve tissue samples were washed with phosphate-buffered saline (PBS), placed into a cryogenic vial, frozen in liquid nitrogen for 10 min, and subsequently preserved at −80 °C.

### Behavioral tests

On day 1 before surgery and day 7 after surgery, the paw withdrawal mechanical threshold (PWMT) and paw withdrawal thermal latency (PWTL) were measured in rats. For PWMT measurement, after the rats had acclimated to the environment, Von Frey filaments were applied perpendicularly to the mid-plantar surface of the hind paw in an ascending order of force until the rat exhibited a withdrawal or escape response. The force value (in grams) at this point was recorded. Each animal was tested five times consecutively, with a 10-min interval between tests (Zhao et al., 2020). For PWTL measurement, after the rats had stabilized, a thermal analgesia meter was used to measure the response of the mid-plantar surface of the left hind paw to thermal stimulation. The PWTL was defined as the time from the onset of irradiation to the appearance of a withdrawal reflex, such as lifting, escaping, or licking the paw. Similarly, each animal was tested five times consecutively, with a 10-min interval between tests (Saadh et al., 2023).

### Protein chip detection

Protein samples were collected from rat nerve tissues and lysed using the Raybiotech kit according to the manufacturer’s instructions. Protein concentrations were determined using the Pierce BCA Protein Assay Kit (Thermo Fisher Scientific, Waltham, MA, USA). Protein chip analysis were using the Rat Cytokine Array 27 (RayBiotech, Inc., GA, USA). The chip included 5 types of cytokines, such as Inflammatory-related factors, cell chemotaxis-related protein factors, neurotrophic-related proteins, cell generation-related protein and vascular endothelial growth factor. More details were in Table 1. The protein chip was included 12 sample wells. The chip sample wells were firstly blocked by sample diluent for 1 h, then the 100 μL protein samples (diluted to 500 μg/mL) were added to completely cover each chip sample well and incubated overnight at 4 °C on a horizontal shaker. After incubation, remove the samples from each well and wash them for six times with Wash Buffers I and II, respectively. Each well was added an 80 μL biotinylated antibody solution and incubated at room temperature on a shaker for 2 h. After the chips were washed, an 80 μL solution of Cy3-streptavidin was added to each well, and the array was incubated in the dark room for 1 h at room temperature, and then rinsed and washed. The chip was scanned at 532 nm using the InnoScan 300 microarray scanner (Innopsys, Inc., Pairs, France).

**Table 1:** Expression levels of 27 cytobines (Raybiotech Rat Cytokine Array 27) on days 1, 3, 7 after CCI vs. the control group.

Category	Proteins	*Genes*	Day 1		Day 3		Day 7	
			Fold change	*p*-value	Fold change	*p*-value	Fold change	*p*-value
Inflammatory-related factors (12)	ICAM-1	*Icam-1*	0.91	0.136	**1.21** ^**#**^	**0.002** ^**^	1.12	0.049
	IFN-gamma	*Ifng*	0.93	0.310	1.06	0.386	0.95	0.484
	IL-10	*Il-10*	1.03	0.646	1.12	0.058	1.00	0.998
	IL-13	*Il-13*	0.99	0.903	1.15	0.068	1.09	0.258
	IL*-*1 α	*Il-1 a*	**1.76** ^**#**^	**<0.001** ^**^	**1.98** ^**#**^	**<0.001** ^**^	**1.37** ^**#**^	**<0.001** ^**^
	IL-1 beta	*Il-1 b*	1.03	0.672	**1.20** ^**#**^	**0.004** ^**^	1.04	0.495
	IL-2	*Il-2*	1.00	0.949	**1.21** ^**#**^	**<0.001** ^**^	1.02	0.700
	IL-4	*Il-4*	0.95	0.481	1.10	0.155	1.02	0.800
	IL-6	*Il-6*	**1.74** ^**#**^	**<0.001** ^**^	**1.86** ^**#**^	**<0.001** ^**^	**2.73** ^**#**^	**<0.001** ^**^
	Prolactin R	*Prlr*	0.90	0.174	**0.82** ^**#**^	**0.006** ^**^	0.87	0.061
	RAGE	*Ager*	1.01	0.862	0.87	0.073	1.13	0.104
	TNFa	*Tnf*	0.99	0.906	**1.20** ^**#**^	**0.005** ^**^	1.03	0.563
cell chemotaxis-related protein factors (10)	B7-2	*Cd86*	1.11	0.081	**1.18** ^**#**^	**0.006** ^**^	**1.24** ^**#**^	**<0.001** ^**^
	CINC-1	*Cxcl1*	**2.61** ^**#**^	**<0.001** ^**^	**2.31** ^**#**^	**<0.001** ^**^	1.14	0.067
	CINC-2	*Cxcl3*	**5.45** ^**#**^	**<0.001** ^**^	**6.28** ^**#**^	**<0.001** ^**^	**4.13** ^**#**^	**<0.001** ^**^
	CINC-3	*Cxcl2*	**4.69** ^**#**^	**<0.001** ^**^	**6.21** ^**#**^	**<0.001** ^**^	**1.85** ^**#**^	**<0.001** ^**^
	Fractalkine	*Cx3cl1*	**0.67** ^**#**^	**<0.001** ^**^	**1.36** ^**#**^	**0.001** ^**^	**0.66** ^**#**^	**<0.001** ^**^
	LIX	*Cxcl5*	**1.52** ^**#**^	**<0.001** ^**^	**2.51** ^**#**^	**<0.001** ^**^	**5.03** ^**#**^	**<0.001** ^**^
	L-Selectin	*Sell*	**1.15** ^**#**^	**0.024** ^*^	**1.45** ^**#**^	**<0.001** ^**^	**1.37** ^**#**^	**<0.001** ^**^
	MCP-1	*CCL-2*	**1.20** ^**#**^	**0.002** ^**^	**1.43** ^**#**^	**<0.001** ^**^	1.05	0.292
	PDGF-AA	*Pdgfa*	**1.24** ^**#**^	**0.002** ^**^	**1.46** ^**#**^	**<0.001** ^**^	**1.15** ^**#**^	**0.030** ^*^
	TCK-1	*Ppbp*	**1.22** ^**#**^	**0.047** ^*^	**1.30** ^**#**^	**0.008** ^**^	1.15	0.140
neurotrophic-related proteins (2)	b-NGF	*Ngf*	**2.63** ^**#**^	**<0.001** ^**^	**1.90** ^**#**^	**<0.001** ^**^	**1.40** ^**#**^	**0.010** ^*^
	CNTF	*Cntf*	**0.67** ^**#**^	**<0.001** ^**^	**0.84** ^**#**^	**0.047** ^*^	**0.79** ^**#**^	**0.008** ^**^
cell generation-related protein factors (2)	GM-CSF	*Csf2*	**1.40** ^**#**^	**<0.001** ^**^	**0.76** ^**#**^	**<0.001** ^**^	1.14	0.047
	TIMP-1	*Timp-1*	**1.22** ^**#**^	**0.003** ^**^	**1.55** ^**#**^	**0.013** ^*^	**1.29** ^**#**^	**0.005** ^**^
vascular endothelial growth factor (1)	VEGF	*Vegfa*	**1.26** ^**#**^	**0.001** ^**^	**1.71** ^**#**^	**<0.001** ^**^	1.02	0.750

The fold change in Table 1 means the ratios of the difference for each protein between the CCI and control group were calculated by the log2.

# indicate the ratios were increased or reduced by more than 15%.

* indicate differences at *p*-value < 0.05, and those with ** indicate differences at *p*-value < 0.01 (moderated *t*-statistic).

### Bioinformatic analysis

The expression levels of proteins at 1, 3, and 7 days post sciatic nerve injury were compared with those in the control group. Proteins with an expression fold change ≥1.5 or ≤0.85 and *p*-value < 0.05 were considered significantly differentially expressed. This analysis was conducted using the moderated *t*-statistics method from the limma package. The differentially expressed proteins (DEPs) were performed by R packages R Project v 4.3.2,^1^ and the cluster heatmap of all DEPs between the control and CCI groups was generated using gplots package of R v 3.1.3.^2^ The Venn diagram analysis was created by Draw Venn Diagram.^3^ KEGG pathway and GO categories were performed using clusterProfiler package of R v 4.6.2 on the differentially proteins using their gene IDs. The *p*-value < 0.05 was used to filter the terms of KEGG and GO enrichment. The terms of related to DEPs in these categories were sorted in ascending order of *P*.adjust, and the top 20 results were selected and plotted as bubble charts. The interconnections between proteins and their functions were visually performed using a Circos plot, created with the BioLadder online analysis software.^4^

### ELLISA detection

Total protein was extracted by lysing the sciatic nerve tissues in a Lysis Buffer solution. The levels of CNIC-2 and CNTF in total protein of these tissues were quantified using RayBio ELISA kits (RayBiotech, Inc., GA, USA), following the manufacturer’s guidelines. Each sample was operated in duplicate within a 96-well plate. A standard curve was generated utilizing lyophilized pure protein to ascertain sample concentrations. The colorimetric change was assessed by measuring absorbance at 450 nm with a Biotek Elx800 microplate reader (Biotek, Inc., Vermont, USA). Concentration values were derived using SigmaPlot 12.0 software and reported in picograms per milliliter. Data analysis was conducted employing GraphPad Prism software (GraphPad Prism, Inc., San Diego, California, USA).

### Statistical analysis

Data analysis was conducted using Excel software (Microsoft, Redmond, WA, USA), with results presented as mean ± standard deviation (SD). The statistical significance was evaluated using the moderated *t*-statistics or Student’s *t*-test. Differences were considered significant if *p*-value < 0.05.

## Results

### Establishment of the CCI model and behavioral results

**Figure 1:**
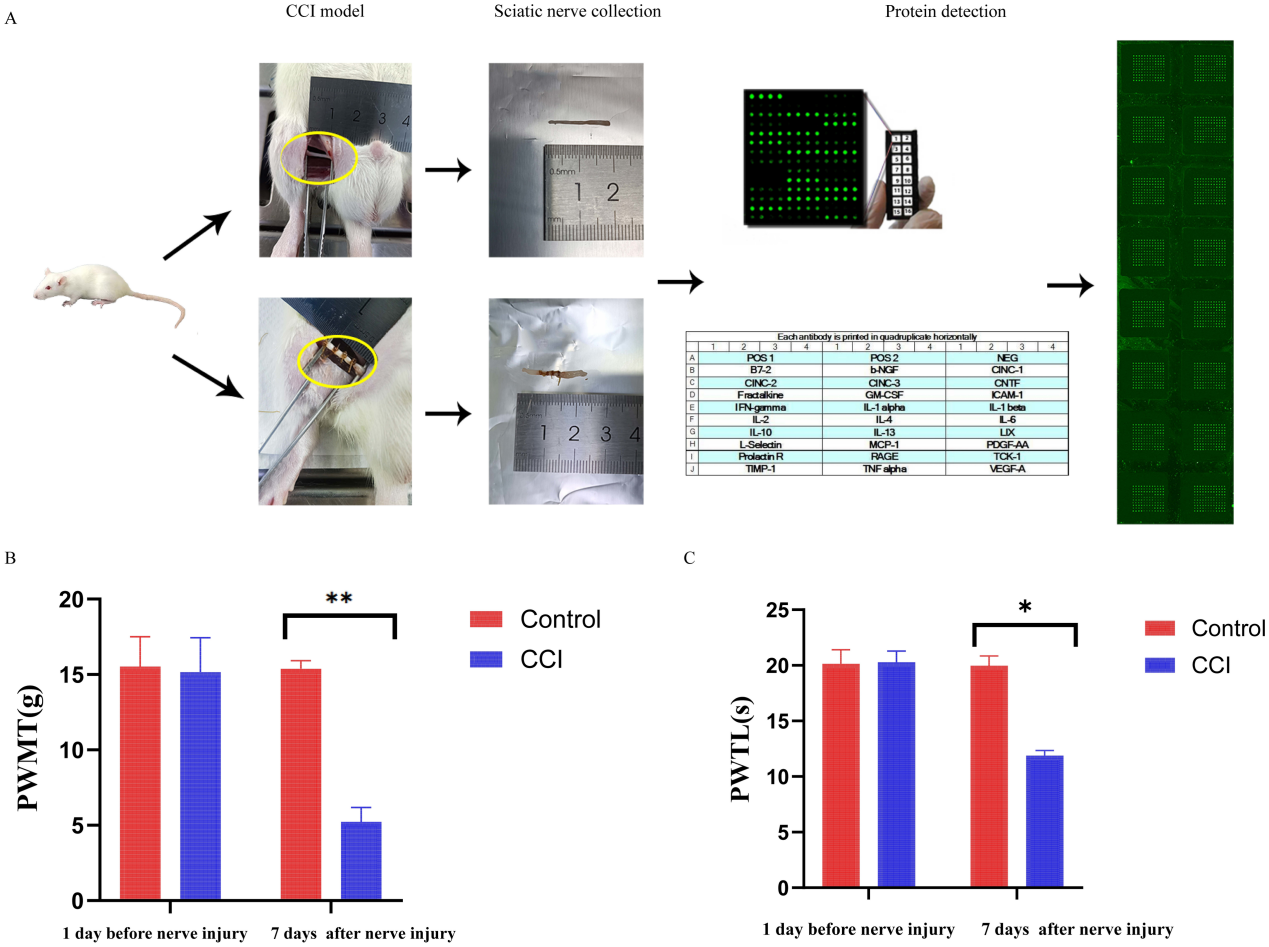
Flowchart of CCI Rat model establishment and behavioral results. **(A)** Study flow diagram. Rat behavioral tests of **(B)** PWMT (*n* = 3/group) and **(C)** PWTL (*n* = 3/group). *indicates *p* < 0.05, and **indicates *p* < 0.01.

In this study, A total of 20 rats were used in this study, with 11 assigned to the CCI group and nine to the control group. To establish the CCI model, the sciatic nerve in the CCI group was ligated with 4.0 chromic gut suture. In contrast, the control group underwent identical surgical exposure of the sciatic nerve without ligation or other manipulation. Nerve tissues were collected from three rats in each of the control and CCI groups at 1, 3, and 7 days post-surgery for further examination. Subsequently, protein samples were extracted from the sciatic nerve tissues and analyzed via protein chips to detect the expression levels of 27 factors associated with sciatic nerve pain (Figure 1A). Additionally, we further evaluated nociceptive behavioral changes in both groups of rats by measuring the paw withdrawal threshold and thermal paw withdrawal latency. The results revealed that compared to the day before nerve injury, rats in the CCI model group exhibited significant mechanical allodynia and thermal hyperalgesia 7 days after nerve injury (Figures 1B,C).

### Proteomic changes during early-stage sciatic nerve injury progression

**Figure 2:**
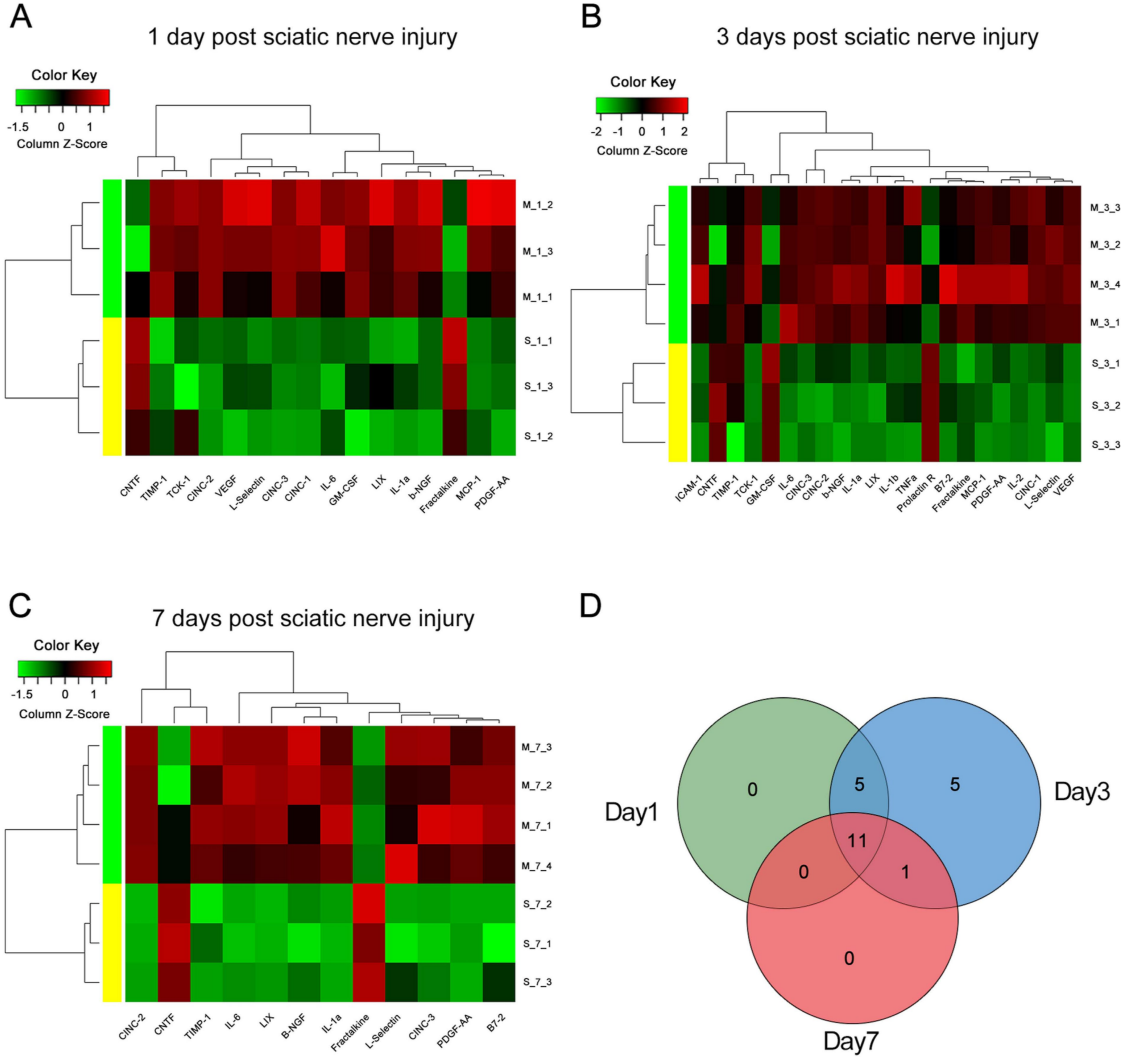
The cluster heatmap and venn diagram of all DEPs between the CCI and control group. **(A–C)** The cluster heatmap analysis delineating the expression patterns of proteins at **(A)** 1 day (*n* = 3), **(B)** 3 days (*n* = 4), and **(C)** 7 days (*n* = 4) post sciatic nerve injury compared with the control group’s profiles. DEPs were defined by a *p-*value < 0.05 (Student’s *t*-test) and a fold change of ≥1.5 or ≤0.85. Red indicates higher expression, while green indicates lower expression. **(D)** Venn diagram showing the number of DEPs at 1 day (*n* = 3), **(B)** 3 days (*n* = 4), and **(C)** 7 days (*n* = 4) post sciatic nerve injury.

To explore the temporal dynamics of protein changes during the progression of early-stage sciatica, we analyzed the DEPs between the CCI and control groups at 1, 3, and 7 days after surgery. On the first day post-injury, 16 proteins were significantly elevated in the CCI group (*p* < 0.05), including key inflammatory mediators (IL-1α, IL-6), neurotrophic factors (*β*-NGF, CNTF), and chemotactic factors (CINC-1, CINC-2, CINC-3, Fractalkine, LIX) (Figure 2A and Table 1). The proteomic landscape evolved by day 3, with 19 proteins significantly up-regulated. Notably, the immunomodulatory protein B7-2, which was not elevated on day 1, emerged as a significantly increased factor at this time point (*p* < 0.05), suggesting a shift in the immune response (Figure 2B and Table 1). By day 7, a more refined set of proteins, including IL-1α, IL-6, B7-2, CINC-2, CINC-3, LIX, L-Selectin, *β*-NGF, and TIMP-1, maintained significant up-regulation (*p*<0.05) (Figure 2C and Table 1). To delineate the temporal expression patterns, we categorized the DEPs based on their persistence across time points. A core group of proteins was significantly up-regulated at all three time points (Figure 2D). This included six chemotaxis-related factors (CINC-2, CINC-3, Fractalkine, LIX, L-Selectin, PDGF-AA), two central inflammatory cytokines (IL-1α and IL-6), and the tissue remodeling factor TIMP-1, defining a sustained response signature. Among neurotrophic factors, β-NGF was part of this sustained up-regulation profile, while CNTF exhibited a persistent decrease from day 1 onwards. This temporal profiling highlights β-NGF, IL-1α, IL-6, and key chemokines as early-initiating and sustained drivers of the pathology, whereas the emergence of B7-2 marks a secondary phase of immune activation.

### KEGG enrichment analysis screened important signaling pathways

**Figure 3:**
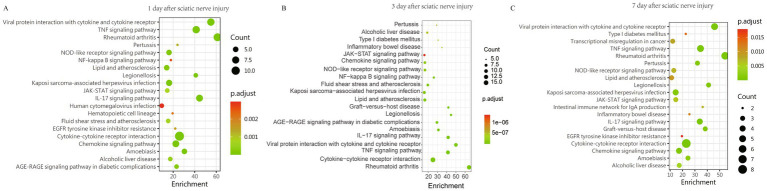
KEGG enrichment analysis of DEPs between the CCI and control group at 1, 3, 7 days post sciatic nerve injury. **(A–C)** The enrichment signal pathways involved in DEPs at **(A)** 1 day (*n* = 3), **(B)** 3 days (*n* = 4), and **(C)** 7 days (*n* = 4) post sciatic nerve injury. The top 20 pathways with *p-*value < 0.05 are listed. The *Y*-axis means the signal pathways, and the *X*-axis means the enrichment score. The size of bubbles indicates the number of DEPs. The color of the bubbles changes from green to yellow, and finally to red, and the corresponding enrichment *P.adjust* is larger.

To investigate the potential function of these DEPs in the early-stage sciatica, we performed KEGG pathway analyses. Top 20 KEGG enrichment analysis results of DEPs in CCI compared with control group were shown in Figure 3 and Supplementary Table 1. The results indicate that on the 1, 3, and 7 days following sciatic nerve injury, the upregulated proteins in the CCI group were predominantly enriched in the Cytokine-cytokine receptor interaction, IL-17 signaling pathway, Chemokine signaling pathway, TNF signaling pathway, NOD-like receptor signaling pathway, JAK–STAT signaling pathway, and NF-kappa B signaling pathway (Figures 3A–C). These findings suggest that the binding of cytokines to their receptors initiates a series of signal transduction pathways associated with validation, thereby triggering an inflammatory response that promotes the onset and sustained progression of early sciatica.

### GO functional enrichment analysis reveals the roles of DEPs in early-stage sciatica

**Figure 4:**
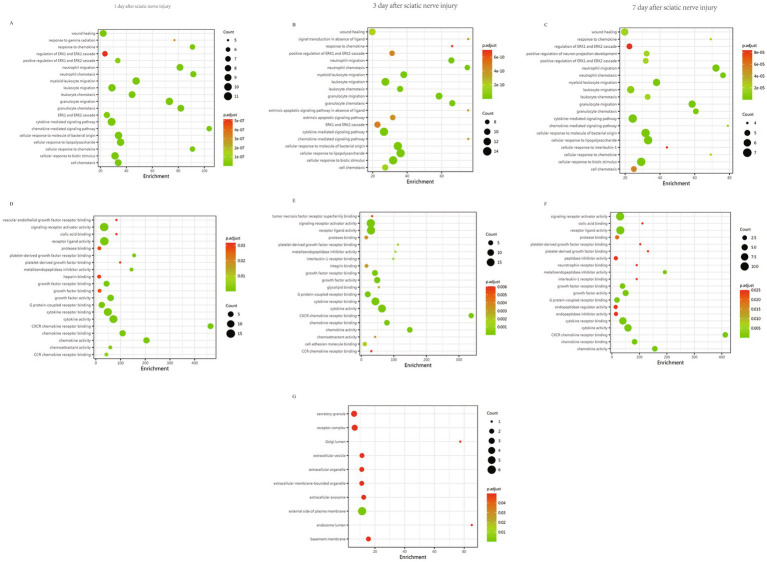
GO classification of DEPs between the CCI and control group at 1, 3, 7 days post sciatic nerve injury. **(A–C)** GO analysed the biological processes (BP) associated with the DEPs between the CCI and control group at **(A)** 1 day (*n* = 3), **(B)** 3 days (*n* = 4), and **(C)** 7 days (*n* = 4) post sciatic nerve injury. **(D–F)** GO analysed the molecular functions (MF) associated with the DEPs between the CCI and control group at **(D)** 1 day (*n* = 3), **(E)** 3 days (*n* = 4), and **(F)** 7 days (*n* = 4) post sciatic nerve injury. **(G)** GO analysed the cellular components (CC) associated with the DEPs between the CCI and control group at 3 days (*n* = 4) post sciatic nerve injury. The top 20 biological processes/molecular functions/cellular components with *p*-value < 0.05 were listed. The *Y*-axis indicates the terms in BP, MF, CC and the *X*-axis indicates the enrichment score. The size of bubbles indicates the number of DEPs in different terms. The color of the bubbles changes from green to yellow, and finally to red, and the corresponding enrichment *P.adjust* is larger.

To further understand the functions of DEPs, they were analyzed based on GO enrichment analysis. The GO system comprises three key functional categories: Biological Process (BP), Molecular Function (MF), and Cellular Component (CC). The top 20 GO terms related DEPs in biological process were list in Figure 4 and Supplementary Table 2. On days 1–7 post CCI, biological processes related to immune response, cellular chemotaxis and inflammation were activated. These include neutrophil/leukocyte chemotaxis and migration, cellular responses to biological stimuli or bacterial-derived molecules (such as lipopolysaccharides), and wound healing, etc. Cytokine-mediated signaling and ERK1/2 cascades were enhanced. Apoptosis-related pathways were activated on day 3 post CCI, including exogenous apoptotic signaling pathways, exogenous apoptotic signaling pathways in the absence of ligands, and ligand-independent signaling pathways. On the 7th day, the positive regulation of neuronal projection development was enhanced. MF enrichment analysis shown that DEPs were involved in critical functions such as chemokine-receptor binding, regulating receptor ligand and signal receptor activity, sialic acid binding, PDGF receptor binding, neurotrophic factor receptor binding, metallopeptidase activity modulation, and IL-1 receptor binding (Figures 4D–F, Supplementary Table 3). This implies DEPs maybe participate in cell recognition, immune responses, vascular tissue fibrosis, and neuroinflammation, aiding the body in responding to sciatic nerve injury. CC enrichment analysis indicates DEPs possibly associated with the plasma membrane’s extracellular side, basement membrane, receptor complexes, extracellular vesicles/organelles, Golgi lumen, and secretory granules (Figure 4G, Supplementary Table 4). Post-sciatic nerve injury may lead to enhanced protein processing and secretion in the Golgi apparatus. These proteins may be cytokines that bind to membrane receptors to form complexes, mediating intracellular signal transduction.

At early-stage sciatica, the Golgi apparatus enhances protein processing and secretion. Chemokines (e.g., CXCL1/2/3/5, CCL2) bind to extracellular chemokine receptors (CXCR/CCR), transmitting signals intracellularly. This recruits immune cells (neutrophils, monocytes) to the injury site, initiating an immune response. Enrichment of responses to LPS and bacterial molecules indicates that in the early-stage of sciatic nerve injury, there may be microbial-related molecular pattern recognition in peripheral nerves, triggering an innate immune response.

### Functional analysis of key DEPS

**Figure 5:**
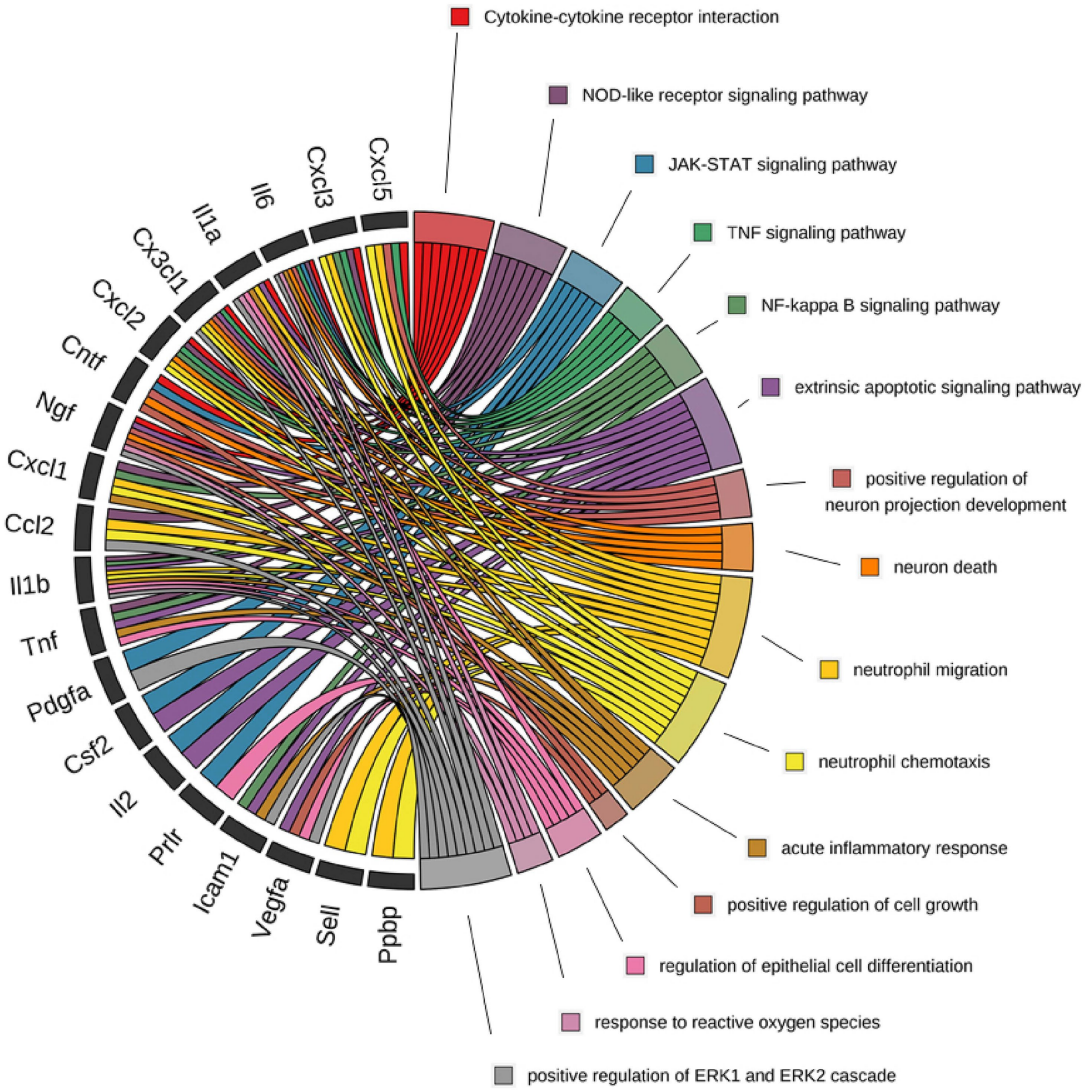
The relationship between the DEPs and functional terms (biological processes and canonical pathways) post sciatic nerve injury. Multiple biological processes and canonical pathways are labeled by different colors in circles on the right. The proteins linked to each term are listed in circles on the left.

To further explore the functions of proteins, we conducted a correlation analysis between differentially expressed proteins and their functions, the results of which are shown in Figure 5. Following sciatic nerve injury, the expression levels of inflammatory factors (IL-1α, IL-6, TNF, IL-1β) and chemokines (CXCL1/2/3, CCL-2) increase. These cytokines, in addition to participating in the transduction of various signaling pathways such as the NOD-like receptor signaling pathway and the NF-κB signaling pathway, also promote the chemotaxis and migration of neutrophils, generating an immune response, thereby enhancing the inflammatory state within the neural tissue. VEGF may be involved in the regulation of cell differentiation and growth. Neurotrophic factors such as CNTF and β-NGF are primarily involved in the regulation of neurite development, neuronal apoptosis, and related pathways. Moreover, studies have found that sciatic nerve pain leads to increased levels of oxidative stress in the body, resulting in the production of a large amount of reactive oxygen species (ROS). This process affects the expression of inflammatory factors such as IL-6, IL-1α, IL-1β, and the neurotrophic factor β-NGF.

### Validation of key protein expression by ELISA

**Figure 6:**
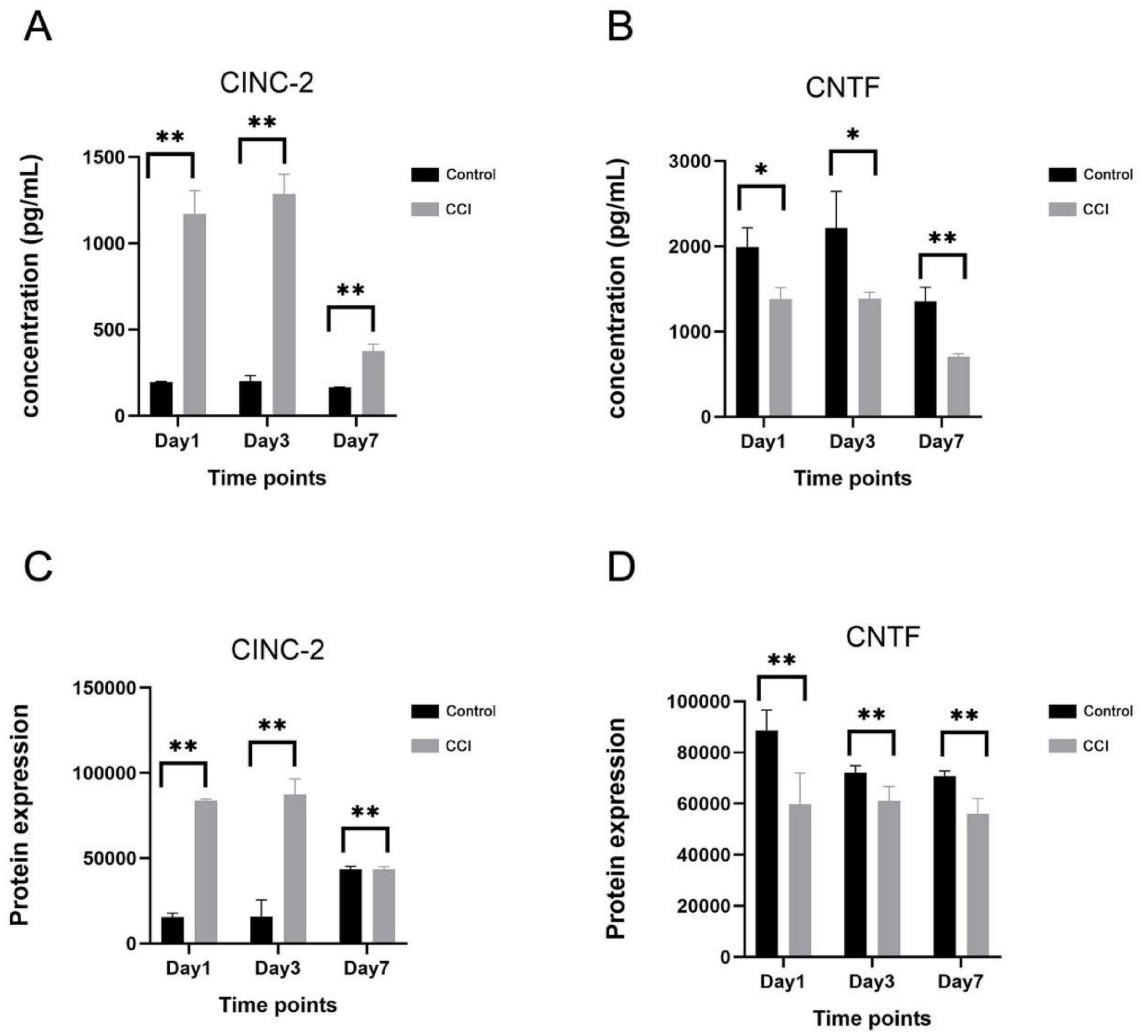
Quantitative analysis of the cytokines CINC-2 and CNTF in sciatic nerve tissue. **(A,B)** ELISA determined the concentrations of CINC-2 and CNTF in sciatic nerve tissues between the CCI and control groups at 1 day (*n* = 3), 3 days (*n* = 4), and 7 days (*n* = 4) post sciatic nerve injury. Data represent the mean ± SD (*n* = 3). *p < 0.05, **p < 0.01(Student’s *t*-test). **(C,D)** Protein microarray was used to evaluated CINC-2 and CNTF expression levels between the CCI and control group at 1 day (*n* = 3), 3 days (*n* = 4), and 7 days (*n* = 4) post sciatic nerve injury using analysis. *p < 0.05, **p < 0.01 (moderated *t*-statistic).

The results of this study indicate that during the early development stage of sciatica, the expression levels of cell chemotaxis-related protein factor CINC-2 significantly increased, suggesting its involvement in the disease process by regulating inflammatory responses. Concurrently, the neuroprotective factor CNTF showed a marked downtrend in the model group. Previous studies have reported that reduced CNTF expression can exacerbate nerve damage. Based on these findings, this study selected CINC-2 and CNTF, two key regulatory factors, for subsequent experimental validation. ELISA was performed to detect the protein concentrations of CINC-2 and CNTF at 1, 3, 7 days post sciatic nerve injury. The results showed that compared with the control group, the expression level of CINC-2 in the CCI group was significantly increased at day 1, 3 and 7, while the expression level of CNTF was continuously decreased (Figures 6A,B). The above results were consistent with those of microarray protein chip (Figures 6C,D).

## Discussion

Sciatica is a common occurrence which is marked by motor, sensory and autonomic dysfunction well and along with pathological neuropathic pain. Chronic neuroinflammation results in neuropathic pain (Ji et al., 2016). In research models, the chronic constriction injury (CCI) model induces only mild damage to the sciatic nerve, with relatively fewer axonal injuries compared to the sciatic nerve compression injury (SCNCI) model, which results in axonal rupture and severe anatomical damage (Wei et al., 2022). The inflammatory response elicited in the CCI model plays a critical role in triggering pathological neuropathic pain (Stevens et al., 2019). Consequently, the CCI model is considered more appropriate for investigating the molecular mechanism of sciatica. Serum samples reflect systemic inflammatory states (Thachil et al., 2024). As the direct site of sciatica, the sciatic nerve tissue can show specific expression changes of key mechanism molecules (Kui et al., 2025; Wei et al., 2023). In this study, based on protein microarray technology, we identified several key inflammatory factors and neurotrophic-related proteins that exhibited differential changes in the CCI model group at 1, 3, 7 days following sciatic nerve injury. Through the analysis of multiple signaling pathways, we elucidated the involvement of various signaling pathways (such as the Cytokine-cytokine receptor interaction, Chemokine, TNF, NOD-like receptor, and NF-kappa B signaling pathways) in the progression of early sciatica. These findings not only provide a comprehensive understanding of the molecular mechanisms underlying early sciatica but also offer potential therapeutic targets for the management of sciatic nerve injury and neuropathic pain.

The imbalance of inflammatory cytokines is associated with pain generation. Various proinflammatory cytokines, such as IL-1β, TNF-α, and IL-6, are associated with peripheral sensitization (Zhang et al., 2022b; Li et al., 2023). The serum concentrations of IL-6 and TNF-α are closely related to the severity of pain and functional impairment in sciatica (Wang et al., 2016). CCI induced an increase in IL-1α, and IL-6 levels in sciatic nerve tissue. On day 3 after CCI surgery, the levels of TNF-α, IL-1β, and IL-2 were elevated. This study detected significantly upregulated IL-1α expression on day 1 of CCI. IL-1α exists as a biologically active precursor in all healthy tissues, whereas IL-1β is produced by myeloid cells during inflammation and requires processing by the NLRP3 inflammasome (Cavalli et al., 2021). IL-1α is more responsive to environmental changes. Both IL-1α and IL-1β, can bind to IL-1R1 to form the IL-1R complex, activating key downstream signaling proteins, such as mitogen-activated protein kinase (ERK1/2), thereby regulating the expression of various inflammatory factors and catabolic genes (Li et al., 2023). GO enrichment analysis revealed that post sciatic nerve injury, IL-1α binding to the interleukin-1 receptor was observed. It has been demonstrated that, the activation and expression of inflammatory cytokines not only directly induce neuropathic pain but also participate in the regulation of inflammatory cascades, thereby promoting the occurrence and persistent development of early-stage sciatica.

**Figure 7:**
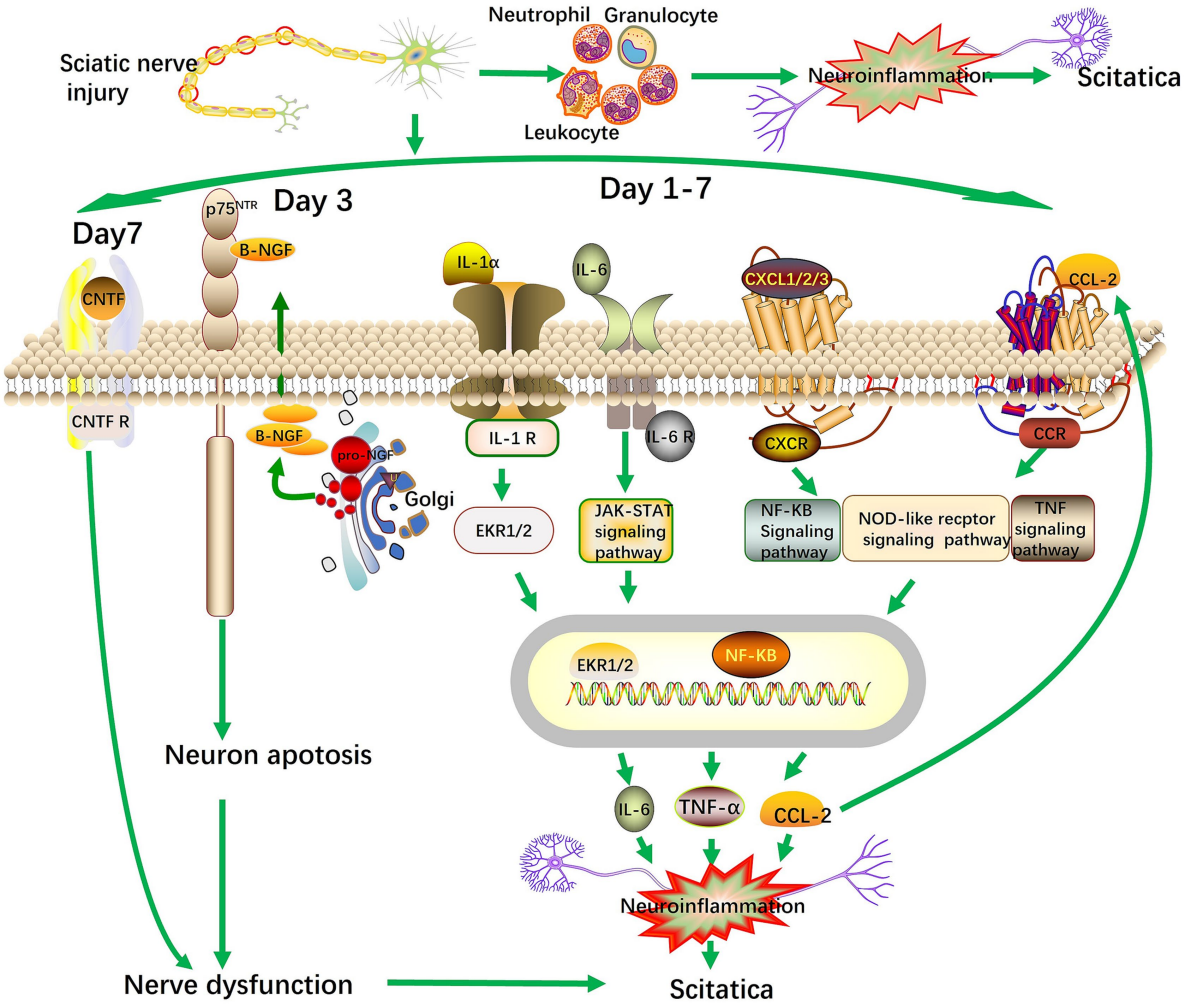
The diagram of the molecular mechanisms of early stage sciatica.

Pathological neuropathic pain is an abnormal interaction between the nervous system and the immune system (Kiguchi et al., 2017). After nerve injury, damaged cells secrete pro-inflammatory molecules that recruit circulating white blood cells to the injury site (Kiguchi et al., 2017; Marchand et al., 2005). Various types of cells, such as microglia, neutrophils, and macrophages are mediated the neuroinflammatory response (Li et al., 2023). In the spared nerve injury (SNI) model, depleting neutrophils in the early injury phase (3–7 days) significantly reduces mechanical allodynia in animals (Nadeau et al., 2011). Our findings indicate that following the sciatic nerve injury, chemokines were significantly upregulated with CXCL1/2/3/5, CCL-2, L-Select, and Pdgfa. High-level expression of cytokine-induced neutrophil chemoattractants CXCL1/2/3/5 and cell adhesion moleculer L-Select enhanced the interaction between leukocytes and the vascular wall, which was key for white blood cells chemotaxis toward the injury site. Among the top 20 biological processes in GO enrichment, the chemotaxis and migration of immune cells such as neutrophils and granulocytes, are significantly enriched. It was speculated that the accumulation of neutrophils and other immune cells at the site of sciatic nerve injury triggers a neuroinflammatory response (Figure 7). In addition to phagocytosis, neutrophils also release pain-causing inflammatory mediators such as cytokines IL-6, TNF-α and chemokine CCL-2, initiating macrophage infiltration and activation (Huerta et al., 2025). CCL2 (C-C motif chemokine ligand 2) is crucial for hyperalgesia post-neural injury (Kwiatkowski et al., 2017; Thacker et al., 2009). We speculate that in the early-stages of sciatica, the accumulation of neutrophils and other immune cells released inflammatory mediators like IL-6, TNF-α, and CCL-2 at the injury site. These cytokines bind to ligands on cell membranes, causing sustained neuronal inflammation, exacerbating nerve damage and driving the progression of sciatica (Figure 7).

Sciatic neuropathic pain is closely related to the abnormal expression of nerve growth factor β-NGF. β-NGF levels in the serum of clinical neuropathic pain patients are upregulated (Monteleone et al., 2018). The application of β-NGF antibodies significantly alleviate SNL-and CCI-induced neuropathic pain (Miyagi et al., 2016). Previous studies have confirmed that the neurotrophic factor CNTF has a significant protective effect on nerve cells (Muhammad et al., 2025). Under pathological conditions such as nerve injury or neurodegenerative diseases, CNTF can promote the survival, differentiation, and regeneration of nerve cells, reduce neuroinflammatory responses, and maintain the stability of nerve function by activating its receptors and related signaling pathways (Cao et al., 2010; Do Rhee et al., 2022). However, when the level of CNTF is reduced, this protective mechanism of nerve cells is weakened, making nerve cells more susceptible to the effects of injury factors, thereby exacerbating nerve damage and impeding the recovery of nerve function. In this study, after CCI in rat, the protein level of β-NGF in the sciatic nerve tissue significantly increased within 7 days, while CNTF significantly decreased. The abnormal expression of β-NGF is one of the causes of early-stage sciatica. Mature NGF is processed from the immature precursor proNGF in the Golgi apparatus (Tramontano et al., 2011; Pagadala et al., 2006). β-NGF has a high-affinity receptor TrkA and a low-affinity receptor p75NTR which is a member of the TNF receptor superfamily. Through its interaction with these receptors, β-NGF exerts diverse functions (Jaffal and Khalil, 2024). Our results showed that following sciatic nerve injury, upregulated β-NGF was significantly associated with the Golgi lumen and endosome lumen, based on GO cellular component enrichment analysis. GO molecular function analysis indicate that β-NGF binds to the TNF receptor superfamily, and GO biological process analysis shown its involvement in extrinsic apoptosis pathways, including those without ligand activation. We speculate that sciatic nerve injury promotes the processing of β-NGF into its active form within the Golgi apparatus, followed by its secretion into endosomes. β-NGF then induces neuronal apoptosis by binding to p75NTR receptors, thereby causing neural dysfunction in the early-stages of sciatica. Meanwhile, the persistent decrease in CNTF leads to aggravated nerve injury, thereby promoting the progression of early sciatica (Figure 7). Several limitations should be acknowledged. First, our study focused on the early phase (7 days), while chronic sciatica may involve distinct mechanisms. Second, while we identified protein changes, causal relationships require functional validation. Third, the CCI model primarily reflects inflammatory components of sciatica rather than compressive etiologies. Finally, the absence of intermediate behavioral monitoring at day 3 represents a limitation in mapping the complete temporal progression of pain behavior. Future studies should combine proteomics with behavioral testing across longer timepoints and validate targets using genetic or pharmacological approaches.

## Conclusion

In conclusion, the progression of early stage sciatica is closely associated with neuroinflammation triggered by the overexpression of inflammatory cytokines and chemokines, as well as nerve dysfunction mediated by neurotrophic-related proteins. Experimental validation has confirmed the significant upregulation of key proteins CNTF and CINC-2 in the CCI model of nerve injury. These findings provide valuable insights into the mechanisms underlying the progression of early stage sciatica and identify potential therapeutic targets.

## Data Availability

The original contributions presented in the study are included in the article/Supplementary material, further inquiries can be directed to the corresponding author.

## Footnotes

1 https://cran.r-project.org/bin/windows/base/old/4.3.2

2 https://cran.r-project.org/src/contrib/Archive/gplots/gplots_3.1.3.tar.gz

3 https://bioinformatics.psb.ugent.be/webtools/Venn/

4 https://www.bioladder.cn/web/#/chart/28
