# Supplementary material for: Unraveling proteomic signatures and neuroinflammatory networks in a CCI rat model of early sciatica: insights for neuropathic pain mechanisms
Source: Front Mol Neurosci. 2025 Dec 5;18:1674151. doi: 10.3389/fnmol.2025.1674151 (PMC12714889; doi:10.3389/fnmol.2025.1674151)
Supplement: Supplementary file 1 [file Data_Sheet_1.docx]

**Table S1.** KEGG analysed the signaling pathways associated with the DEPs between the CCI and control group at 1, 3, 7 days post sciatic nerve injury.

| ID | Description | *P* value | | *P.adjust* | Gene ID | | Count | Enrich Score | GeneRatio | BgRatio |
| --- | --- | --- | --- | --- | --- | --- | --- | --- | --- | --- |
| **Day1** | | | | | |  | | | | |
| rno04060 | Cytokine-cytokine receptor interaction | 4.52E-16 | | 3.93E-14 | Cxcl1/Cxcl3/Cxcl2/Il1a/Ngf/Csf2/Cntf/Cx3cl1/Il6/Cxcl5/Ccl2/Ppbp | | 12 | 25.88 | 12/16 | 271/9353 |
| rno05323 | Rheumatoid arthritis | 3.32E-15 | | 1.44E-13 | Cxcl1/Cxcl3/Cxcl2/Il1a/Csf2/Il6/Cxcl5/Vegfa/Ccl2 | | 9 | 61.18 | 9/16 | 86/9353 |
| rno04061 | Viral protein interaction with cytokine and cytokine receptor | 4.03E-13 | | 1.17E-11 | Cxcl1/Cxcl3/Cxcl2/Cx3cl1/Il6/Cxcl5/Ccl2/Ppbp | | 8 | 55.02 | 8/16 | 85/9353 |
| rno04668 | TNF signaling pathway | 4.20E-12 | | 9.13E-11 | Cxcl1/Cxcl3/Cxcl2/Csf2/Cx3cl1/Il6/Cxcl5/Ccl2 | | 8 | 41.38 | 8/16 | 113/9353 |
| rno04657 | IL-17 signaling pathway | 7.52E-11 | | 1.31E-09 | Cxcl1/Cxcl3/Cxcl2/Csf2/Il6/Cxcl5/Ccl2 | | 7 | 44.48 | 7/16 | 92/9353 |
| rno04062 | Chemokine signaling pathway | 9.29E-09 | | 1.35E-07 | Cxcl1/Cxcl3/Cxcl2/Cx3cl1/Cxcl5/Ccl2/Ppbp | | 7 | 22.48 | 7/16 | 182/9353 |
| rno05146 | Amoebiasis | 4.10E-07 | | 5.09E-06 | Cxcl1/Cxcl3/Cxcl2/Csf2/Il6 | | 5 | 30.45 | 5/16 | 96/9353 |
| rno05167 | Kaposi sarcoma-associated herpesvirus infection | 9.34E-07 | | 1.02E-05 | Cxcl1/Cxcl3/Cxcl2/Csf2/Il6/Vegfa | | 6 | 16.24 | 6/16 | 216/9353 |
| rno05134 | Legionellosis | 2.14E-06 | | 2.07E-05 | Cxcl1/Cxcl3/Cxcl2/Il6 | | 4 | 41.02 | 4/16 | 57/9353 |
| rno04621 | NOD-like receptor signaling pathway | 9.44E-06 | | 8.21E-05 | Cxcl1/Cxcl3/Cxcl2/Il6/Ccl2 | | 5 | 16.15 | 5/16 | 181/9353 |
| rno05417 | Lipid and atherosclerosis | 1.90E-05 | | 1.50E-04 | Cxcl1/Cxcl3/Cxcl2/Il6/Ccl2 | | 5 | 13.98 | 5/16 | 209/9353 |
| rno04933 | AGE-RAGE signaling pathway in diabetic complications | 2.11E-05 | | 1.53E-04 | Il1a/Il6/Vegfa/Ccl2 | | 4 | 23.15 | 4/16 | 101/9353 |
| rno04936 | Alcoholic liver disease | 6.60E-05 | | 4.42E-04 | Cxcl1/Cxcl3/Cxcl2/Il6 | | 4 | 17.32 | 4/16 | 135/9353 |
| rno05418 | Fluid shear stress and atherosclerosis | 0.000105 | | 6.51E-04 | Il1a/Vegfa/Ccl2/Pdgfa | | 4 | 15.38 | 4/16 | 152/9353 |
| rno04630 | JAK-STAT signaling pathway | 0.000131 | | 7.60E-04 | Csf2/Cntf/Il6/Pdgfa | | 4 | 14.52 | 4/16 | 161/9353 |
| ID | Description | *P* value | *P.adjust* | | Gene ID | | Count | Enrich Score | GeneRatio | BgRatio |
| rno05133 | Pertussis | 0.000237495 | 1.29E-03 | | Il1a/Il6/Cxcl5 | | 3 | 24.02 | 3/16 | 73/9353 |
| rno01521 | EGFR tyrosine kinase inhibitor resistance | 0.000311 | 1.59E-03 | | Il6/Vegfa/Pdgfa | | 3 | 21.92 | 3/16 | 80/9353 |
| rno04640 | Hematopoietic cell lineage | 0.000441 | 2.13E-03 | | Il1a/Csf2/Il6 | | 3 | 19.49 | 3/16 | 90/9353 |
| rno04064 | NF-kappa B signaling pathway | 0.000566 | 2.59E-03 | | Cxcl1/Cxcl3/Cxcl2 | | 3 | 17.89 | 3/16 | 98/9353 |
| rno05163 | Human cytomegalovirus infection | 0.000683 | 2.97E-03 | | Cx3cl1/Il6/Vegfa/Ccl2 | | 4 | 9.43 | 4/16 | 248/9353 |
| **Day3** |  |  |  | |  | |  |  |  |  |
| rno05323 | Rheumatoid arthritis | 6.07E-22 | 6.68E-20 | | Cxcl3/Cxcl2/Cxcl1/Il1a/Cxcl5/Vegfa/Ccl2/Il6/Csf2/Icam1/Il1b/Tnf/Cd86 | | 13 | 64.26 | 13/22 | 86/9353 |
| rno04060 | Cytokine-cytokine receptor interaction | 1.02E-20 | 5.59E-19 | | Cxcl3/Cxcl2/Cxcl1/Il1a/Cxcl5/Ccl2/Ngf/Il6/Csf2/Il2/Cx3cl1/Il1b/Tnf/Prlr/Ppbp/Cntf | | 16 | 25.10 | 16/22 | 271/9353 |
| rno04668 | TNF signaling pathway | 3.08E-16 | 1.13E-14 | | Cxcl3/Cxcl2/Cxcl1/Cxcl5/Ccl2/Il6/Csf2/Cx3cl1/Icam1/Il1b/Tnf | | 11 | 41.38 | 11/22 | 113/9353 |
| rno04061 | Viral protein interaction with cytokine and cytokine receptor | 1.32E-15 | 3.63E-14 | | Cxcl3/Cxcl2/Cxcl1/Cxcl5/Ccl2/Il6/Il2/Cx3cl1/Tnf/Ppbp | | 10 | 50.02 | 10/22 | 85/9353 |
| rno04657 | IL-17 signaling pathway | 2.59E-13 | 5.70E-12 | | Cxcl3/Cxcl2/Cxcl1/Cxcl5/Ccl2/Il6/Csf2/Il1b/Tnf | | 9 | 41.59 | 9/22 | 92/9353 |
| rno05146 | Amoebiasis | 1.45E-09 | 2.65E-08 | | Cxcl3/Cxcl2/Cxcl1/Il6/Csf2/Il1b/Tnf | | 7 | 31.00 | 7/22 | 96/9353 |
| rno04933 | AGE-RAGE signaling pathway in diabetic complications | 2.07E-09 | 3.26E-08 | | Il1a/Vegfa/Ccl2/Il6/Icam1/Il1b/Tnf | | 7 | 29.46 | 7/22 | 101/9353 |
| rno05134 | Legionellosis | 2.71E-09 | 3.72E-08 | | Cxcl3/Cxcl2/Cxcl1/Il6/Il1b/Tnf | | 6 | 44.75 | 6/22 | 57/9353 |
| rno05332 | Graft-versus-host disease | 4.12E-09 | 5.03E-08 | | Il1a/Il6/Il2/Il1b/Tnf/Cd86 | | 6 | 41.82 | 6/22 | 61/9353 |
| rno05417 | Lipid and atherosclerosis | 1.33E-08 | 1.46E-07 | | Cxcl3/Cxcl2/Cxcl1/Ccl2/Il6/Icam1/Il1b/Tnf | | 8 | 16.27 | 8/22 | 209/9353 |
| rno05167 | Kaposi sarcoma-associated herpesvirus infection | 1.72E-08 | 1.72E-07 | | Cxcl3/Cxcl2/Cxcl1/Vegfa/Il6/Csf2/Icam1/Cd86 | | 8 | 15.75 | 8/22 | 216/9353 |
| ID | Description | *P* value | *P.adjust* | | Gene ID | | Count | Enrich Score | GeneRatio | BgRatio |
| rno05418 | Fluid shear stress and atherosclerosis | 3.62E-08 | 3.32E-07 | | Il1a/Vegfa/Ccl2/Pdgfa/Icam1/Il1b/Tnf | | 7 | 19.58 | 7/22 | 152/9353 |
| rno04064 | NF-kappa B signaling pathway | 7.39E-08 | 6.25E-07 | | Cxcl3/Cxcl2/Cxcl1/Icam1/Il1b/Tnf | | 6 | 26.03 | 6/22 | 98/9353 |
| rno04621 | NOD-like receptor signaling pathway | 1.21E-07 | 9.20E-07 | | Cxcl3/Cxcl2/Cxcl1/Ccl2/Il6/Il1b/Tnf | | 7 | 16.44 | 7/22 | 181/9353 |
| rno04062 | Chemokine signaling pathway | 1.25E-07 | 9.80E-07 | | Cxcl3/Cxcl2/Cxcl1/Cxcl5/Ccl2/Cx3cl1/Ppbp | | 7 | 16.35 | 7/22 | 182/9353 |
| rno05144 | Malaria | 1.43E-07 | 7.37E-06 | | Ccl2/Il6/Icam1/Il1b/Tnf | | 5 | 38.65 | 5/22 | 55/9353 |
| rno05321 | Inflammatory bowel disease | 2.42E-07 | 1.56E-06 | | Il1a/Il6/Il2/Il1b/Tnf | | 5 | 34.85 | 5/22 | 61/9353 |
| rno04940 | Type I diabetes mellitus | 4.51E-07 | 2.75E-06 | | Il1a/Il2/Il1b/Tnf/Cd86 | | 5 | 30.81 | 5/22 | 69/9353 |
| rno04936 | Alcoholic liver disease | 4.99E-07 | 2.89E-06 | | Cxcl3/Cxcl2/Cxcl1/Il6/Il1b/Tnf | | 6 | 18.89 | 6/22 | 135/9353 |
| rno05133 | Pertussis | 5.99E-07 | 3.29E-06 | | Il1a/Cxcl5/Il6/Il1b/Tnf | | 5 | 29.12 | 5/22 | 73/9353 |
| **Day7** |  |  |  | |  | |  |  |  |  |
| rno04060 | Cytokine-cytokine receptor interaction | 2.01E-10 | 1.59E-08 | | Cxcl5/Cxcl3/Il6/Il1a/Cx3cl1/Cxcl2/Cntf/Ngf | | 8 | 23.01 | 8/12 | 271/9353 |
| rno05323 | Rheumatoid arthritis | 4.48E-10 | 1.77E-08 | | Cxcl5/Cxcl3/Il6/Il1a/Cxcl2/Cd86 | | 6 | 54.38 | 6/12 | 86/9353 |
| rno04061 | Viral protein interaction with cytokine and cytokine receptor | 4.15E-08 | 1.09E-06 | | Cxcl5/Cxcl3/Il6/Cx3cl1/Cxcl2 | | 5 | 45.85 | 5/12 | 85/9353 |
| rno04668 | TNF signaling pathway | 1.74E-07 | 3.44E-06 | | Cxcl5/Cxcl3/Il6/Cx3cl1/Cxcl2 | | 5 | 34.49 | 5/12 | 113/9353 |
| rno04657 | IL-17 signaling pathway | 4.09E-06 | 6.46E-05 | | Cxcl5/Cxcl3/Il6/Cxcl2 | | 4 | 33.89 | 4/12 | 92/9353 |
| rno05134 | Legionellosis | 4.54E-05 | 5.98E-04 | | Cxcl3/Il6/Cxcl2 | | 3 | 41.02 | 3/12 | 57/9353 |
| rno05332 | Graft-versus-host disease | 5.57E-05 | 6.00E-04 | | Il6/Il1a/Cd86 | | 3 | 38.33 | 3/12 | 61/9353 |
| rno04062 | Chemokine signaling pathway | 6.08E-05 | 6.00E-04 | | Cxcl5/Cxcl3/Cx3cl1/Cxcl2 | | 4 | 17.13 | 4/12 | 182/9353 |
| rno05133 | Pertussis | 9.54E-05 | 8.38E-04 | | Cxcl5/Il6/Il1a | | 3 | 32.03 | 3/12 | 73/9353 |
| rno05167 | Kaposi sarcoma-associated herpesvirus infection | 0.000118 | 9.35E-04 | | Cxcl3/Il6/Cxcl2/Cd86 | | 4 | 14.43 | 4/12 | 216/9353 |
| rno05146 | Amoebiasis | 0.000216 | 1.55E-03 | | Cxcl3/Il6/Cxcl2 | | 3 | 24.36 | 3/12 | 96/9353 |
| ID | Description | *P* value | *P.adjust* | | Gene ID | | Count | Enrich Score | GeneRatio | BgRatio |
| rno04936 | Alcoholic liver disease | 0.000588 | 3.87E-03 | | Cxcl3/Il6/Cxcl2 | | 3 | 17.32 | 3/12 | 135/9353 |
| rno04630 | JAK-STAT signaling pathway | 0.000983 | 5.97E-03 | | Il6/Cntf/Pdgfa | | 3 | 14.52 | 3/12 | 161/9353 |
| rno04672 | Intestinal immune network for IgA production | 0.001323 | 7.26E-03 | | Il6/Cd86 | | 2 | 36.25 | 2/12 | 43/9353 |
| rno04621 | NOD-like receptor signaling pathway | 0.001379 | 7.26E-03 | | Cxcl3/Il6/Cxcl2 | | 3 | 12.92 | 3/12 | 181/9353 |
| rno05202 | Transcriptional misregulation in cancer | 0.001684 | 8.31E-03 | | Il6/Cd86/Pdgfa | | 3 | 12.05 | 3/12 | 194/9353 |
| rno05417 | Lipid and atherosclerosis | 0.002085 | 9.69E-03 | | Cxcl3/Il6/Cxcl2 | | 3 | 11.19 | 3/12 | 209/9353 |
| rno05321 | Inflammatory bowel disease | 0.002648 | 1.16E-02 | | Il6/Il1a | | 2 | 25.55 | 2/12 | 61/9353 |
| rno04940 | Type I diabetes mellitus | 0.003375 | 1.40E-02 | | Il1a/Cd86 | | 2 | 22.59 | 2/12 | 69/9353 |
| rno01521 | EGFR tyrosine kinase inhibitor resistance | 0.004511 | 1.78E-02 | | Il6/Pdgfa | | 2 | 19.49 | 2/12 | 80/9353 |

Note: Gene Ratio=The gene amount of the pathway involved by DEPs after CCI is compared to the gene amount of the pathway in the KEGG Database.

BgRatio= the KEGG Database shows the differential gene amount of the corresponding pathway, involving DEPs after CCI/total proteins.

**Table S2.** GO analysed the biological processes associated with the DEPs between the CCI and control group at 1, 3, 7 days post sciatic nerve injury.

| ID | Description in GO | *P* value | *P.adjust* | Gene ID | Count | Enrich  Score | GeneRatio | BgRatio |
| --- | --- | --- | --- | --- | --- | --- | --- | --- |
| **Day1** | | | |  | | | | |
| GO:0097530 | granulocyte migration | 1.19E-17 | 1.75E-14 | Cxcl1/Cxcl3/Cxcl2/Il1a/Cx3cl1/Cxcl5/Vegfa/Ccl2/Ppbp/Sell | 10 | 73.13 | 10/16 | 158/18488 |
| GO:0071621 | granulocyte chemotaxis | 2.81E-16 | 1.47E-13 | Cxcl1/Cxcl3/Cxcl2/Cx3cl1/Cxcl5/Vegfa/Ccl2/Ppbp/Sell | 9 | 81.89 | 9/16 | 127/18488 |
| GO:1990266 | neutrophil migration | 3.02E-16 | 1.47E-13 | Cxcl1/Cxcl3/Cxcl2/Il1a/Cx3cl1/Cxcl5/Ccl2/Ppbp/Sell | 9 | 81.25 | 9/16 | 128/18488 |
| GO:0097529 | myeloid leukocyte migration | 9.55E-16 | 3.49E-13 | Cxcl1/Cxcl3/Cxcl2/Il1a/Cx3cl1/Cxcl5/Vegfa/Ccl2/Ppbp/Sell | 10 | 47.55 | 10/16 | 243/18488 |
| GO:0019221 | cytokine-mediated signaling pathway | 5.19E-15 | 1.52E-12 | Cxcl1/Cxcl3/Cxcl2/Il1a/Csf2/Cntf/Cx3cl1/Il6/Cxcl5/Ccl2/Ppbp | 11 | 28.69 | 11/16 | 443/18488 |
| GO:0030593 | neutrophil chemotaxis | 7.42E-15 | 1.81E-12 | Cxcl1/Cxcl3/Cxcl2/Cx3cl1/Cxcl5/Ccl2/Ppbp/Sell | 8 | 91.52 | 8/16 | 101/18488 |
| GO:0071222 | cellular response to lipopolysaccharide | 1.79E-14 | 3.74E-12 | Cxcl1/Cxcl3/Cxcl2/Il1a/Csf2/Cx3cl1/Il6/Cxcl5/Ccl2/Ppbp | 10 | 35.55 | 10/16 | 325/18488 |
| GO:0071219 | cellular response to molecule of bacterial origin | 2.82E-14 | 5.15E-12 | Cxcl1/Cxcl3/Cxcl2/Il1a/Csf2/Cx3cl1/Il6/Cxcl5/Ccl2/Ppbp | 10 | 33.99 | 10/16 | 340/18488 |
| GO:0071216 | cellular response to biotic stimulus | 6.40E-14 | 1.04E-11 | Cxcl1/Cxcl3/Cxcl2/Il1a/Csf2/Cx3cl1/Il6/Cxcl5/Ccl2/Ppbp | 10 | 31.31 | 10/16 | 369/18488 |
| GO:0030595 | leukocyte chemotaxis | 7.57E-14 | 1.11E-11 | Cxcl1/Cxcl3/Cxcl2/Cx3cl1/Cxcl5/Vegfa/Ccl2/Ppbp/Sell | 9 | 44.44 | 9/16 | 234/18488 |
| GO:0050900 | leukocyte migration | 1.40E-13 | 1.86E-11 | Cxcl1/Cxcl3/Cxcl2/Il1a/Cx3cl1/Cxcl5/Vegfa/Ccl2/Ppbp/Sell | 10 | 28.96 | 10/16 | 399/18488 |
| GO:0070098 | chemokine-mediated signaling pathway | 2.00E-13 | 2.44E-11 | Cxcl1/Cxcl3/Cxcl2/Cx3cl1/Cxcl5/Ccl2/Ppbp | 7 | 103.70 | 7/16 | 78/18488 |
| GO:1990868 | response to chemokine | 5.20E-13 | 5.44E-11 | Cxcl1/Cxcl3/Cxcl2/Cx3cl1/Cxcl5/Ccl2/Ppbp | 7 | 90.88 | 7/16 | 89/18488 |
| GO:1990869 | cellular response to chemokine | 5.20E-13 | 5.44E-11 | Cxcl1/Cxcl3/Cxcl2/Cx3cl1/Cxcl5/Ccl2/Ppbp | 7 | 90.88 | 7/16 | 89/18488 |
| GO:0060326 | cell chemotaxis | 9.09E-13 | 8.86E-11 | Cxcl1/Cxcl3/Cxcl2/Cx3cl1/Cxcl5/Vegfa/Ccl2/Ppbp/Sell | 9 | 33.76 | 9/16 | 308/18488 |
| GO:0042060 | wound healing | 3.87E-11 | 3.53E-09 | Cxcl2/Il1a/Cx3cl1/Il6/Vegfa/Ccl2/Pdgfa/Ppbp/Timp1 | 9 | 22.22 | 9/16 | 468/18488 |
| GO:0070371 | ERK1 and ERK2 cascade | 2.61E-10 | 2.25E-08 | Il1a/Ngf/Csf2/Cx3cl1/Il6/Vegfa/Ccl2/Pdgfa | 8 | 25.05 | 8/16 | 369/18488 |
| ID | Description in GO | *P* value | *P.adjust* | Gene ID | Count | Enrich  Score | GeneRatio | BgRatio |
| GO:0070374 | positive regulation of ERK1 and ERK2 cascade | 6.25E-10 | 5.08E-08 | Il1a/Ngf/Cx3cl1/Il6/Vegfa/Ccl2/Pdgfa | 7 | 33.42 | 7/16 | 242/18488 |
| GO:0010332 | response to gamma radiation | 4.05E-09 | 3.12E-07 | Cxcl1/Cxcl2/Il1a/Cxcl5/Ccl2 | 5 | 77.03 | 5/16 | 75/18488 |
| GO:0070372 | regulation of ERK1 and ERK2 cascade | 7.05E-09 | 5.16E-07 | Il1a/Ngf/Cx3cl1/Il6/Vegfa/Ccl2/Pdgfa | 7 | 23.58 | 7/16 | 343/18488 |
| **Day3** |  |  |  |  |  |  |  |  |
| GO:0071222 | cellular response to lipopolysaccharide | 5.74E-20 | 1.06E-16 | Cxcl3/Cxcl2/Cxcl1/Il1a/Cxcl5/Ccl2/Il6/Csf2/Cx3cl1/Icam1/Il1b/Tnf/Cd86/Ppbp | 14 | 36.20 | 14/22 | 325/18488 |
| GO:0071219 | cellular response to molecule of bacterial origin | 1.09E-19 | 1.06E-16 | Cxcl3/Cxcl2/Cxcl1/Il1a/Cxcl5/Ccl2/Il6/Csf2/Cx3cl1/Icam1/Il1b/Tnf/Cd86/Ppbp | 14 | 34.60 | 14/22 | 340/18488 |
| GO:0071216 | cellular response to biotic stimulus | 3.45E-19 | 2.24E-16 | Cxcl3/Cxcl2/Cxcl1/Il1a/Cxcl5/Ccl2/Il6/Csf2/Cx3cl1/Icam1/Il1b/Tnf/Cd86/Ppbp | 14 | 31.88 | 14/22 | 369/18488 |
| GO:0019221 | cytokine-mediated signaling pathway | 4.51E-18 | 2.19E-15 | Cxcl3/Cxcl2/Cxcl1/Il1a/Cxcl5/Ccl2/Il6/Csf2/Cx3cl1/Il1b/Tnf/Prlr/Ppbp/Cntf | 14 | 26.56 | 14/22 | 443/18488 |
| GO:0097530 | granulocyte migration | 8.12E-18 | 3.16E-15 | Cxcl3/Cxcl2/Cxcl1/Il1a/Cxcl5/Vegfa/Ccl2/Sell/Cx3cl1/Il1b/Ppbp | 11 | 58.51 | 11/22 | 158/18488 |
| GO:0050900 | leukocyte migration | 7.57E-17 | 2.45E-14 | Cxcl3/Cxcl2/Cxcl1/Il1a/Cxcl5/Vegfa/Ccl2/Sell/Cx3cl1/Icam1/Il1b/Tnf/Ppbp | 13 | 27.38 | 13/22 | 399/18488 |
| GO:0071621 | granulocyte chemotaxis | 9.84E-17 | 2.59E-14 | Cxcl3/Cxcl2/Cxcl1/Cxcl5/Vegfa/Ccl2/Sell/Cx3cl1/Il1b/Ppbp | 10 | 66.17 | 10/22 | 127/18488 |
| GO:1990266 | neutrophil migration | 1.07E-16 | 2.59E-14 | Cxcl3/Cxcl2/Cxcl1/Il1a/Cxcl5/Ccl2/Sell/Cx3cl1/Il1b/Ppbp | 10 | 65.65 | 10/22 | 128/18488 |
| GO:0097529 | myeloid leukocyte migration | 1.00E-15 | 2.16E-13 | Cxcl3/Cxcl2/Cxcl1/Il1a/Cxcl5/Vegfa/Ccl2/Sell/Cx3cl1/Il1b/Ppbp | 11 | 38.04 | 11/22 | 243/18488 |
| GO:0030593 | neutrophil chemotaxis | 1.41E-15 | 2.75E-13 | Cxcl3/Cxcl2/Cxcl1/Cxcl5/Ccl2/Sell/Cx3cl1/Il1b/Ppbp | 9 | 74.88 | 9/22 | 101/18488 |
| GO:0030595 | leukocyte chemotaxis | 4.93E-14 | 8.71E-12 | Cxcl3/Cxcl2/Cxcl1/Cxcl5/Vegfa/Ccl2/Sell/Cx3cl1/Il1b/Ppbp | 10 | 35.91 | 10/22 | 234/18488 |
| GO:0060326 | cell chemotaxis | 7.71E-13 | 1.25E-10 | Cxcl3/Cxcl2/Cxcl1/Cxcl5/Vegfa/Ccl2/Sell/Cx3cl1/Il1b/Ppbp | 10 | 27.28 | 10/22 | 308/18488 |
| GO:0042060 | wound healing | 1.34E-12 | 2.00E-10 | Cxcl2/Il1a/Vegfa/Ccl2/Pdgfa/Il6/Cx3cl1/Il1b/Tnf/Ppbp/Timp1 | 11 | 19.75 | 11/22 | 468/18488 |
| ID | Description in GO | *P* value | *P.adjust* | Gene ID | Count | Enrich  Score | GeneRatio | BgRatio |
| GO:0038034 | signal transduction in absence of ligand | 2.93E-12 | 3.56E-10 | Il1a/Ngf/Csf2/Il2/Cx3cl1/Il1b/Tnf | 7 | 75.42 | 7/22 | 78/18488 |
| GO:0070098 | chemokine-mediated signaling pathway | 2.93E-12 | 3.56E-10 | Cxcl3/Cxcl2/Cxcl1/Cxcl5/Ccl2/Cx3cl1/Ppbp | 7 | 75.42 | 7/22 | 78/18488 |
| GO:0097192 | extrinsic apoptotic signaling pathway in absence of ligand | 2.93E-12 | 3.56E-10 | Il1a/Ngf/Csf2/Il2/Cx3cl1/Il1b/Tnf | 7 | 75.42 | 7/22 | 78/18488 |
| GO:0097191 | extrinsic apoptotic signaling pathway | 3.73E-12 | 4.26E-10 | Il1a/Vegfa/Ngf/Csf2/Il2/Cx3cl1/Icam1/Il1b/Tnf | 9 | 31.65 | 9/22 | 239/18488 |
| GO:0070374 | positive regulation of ERK1 and ERK2 cascade | 4.17E-12 | 4.51E-10 | Il1a/Vegfa/Ccl2/Pdgfa/Ngf/Il6/Cx3cl1/Icam1/Il1b | 9 | 31.25 | 9/22 | 242/18488 |
| GO:0070371 | ERK1 and ERK2 cascade | 4.64E-12 | 4.75E-10 | Il1a/Vegfa/Ccl2/Pdgfa/Ngf/Il6/Csf2/Cx3cl1/Icam1/Il1b | 10 | 22.77 | 10/22 | 369/18488 |
| GO:1990868 | response to chemokine | 7.58E-12 | 7.02E-10 | Cxcl3/Cxcl2/Cxcl1/Cxcl5/Ccl2/Cx3cl1/Ppbp | 7 | 66.10 | 7/22 | 89/18488 |
| **Day7** |  |  |  |  |  |  |  |  |
| GO:1990266 | neutrophil migration | 8.74E-11 | 9.61E-08 | Cxcl5/Cxcl3/Il1a/Cx3cl1/Sell/Cxcl2 | 6 | 72.22 | 6/12 | 128/18488 |
| GO:0097530 | granulocyte migration | 3.14E-10 | 1.31E-07 | Cxcl5/Cxcl3/Il1a/Cx3cl1/Sell/Cxcl2 | 6 | 58.51 | 6/12 | 158/18488 |
| GO:0071222 | cellular response to lipopolysaccharide | 3.57E-10 | 1.31E-07 | Cxcl5/Cxcl3/Il6/Il1a/Cx3cl1/Cxcl2/Cd86 | 7 | 33.18 | 7/12 | 325/18488 |
| GO:0071219 | cellular response to molecule of bacterial origin | 4.90E-10 | 1.35E-07 | Cxcl5/Cxcl3/Il6/Il1a/Cx3cl1/Cxcl2/Cd86 | 7 | 31.72 | 7/12 | 340/18488 |
| GO:0071216 | cellular response to biotic stimulus | 8.67E-10 | 1.91E-07 | Cxcl5/Cxcl3/Il6/Il1a/Cx3cl1/Cxcl2/Cd86 | 7 | 29.23 | 7/12 | 369/18488 |
| GO:0019221 | cytokine-mediated signaling pathway | 3.09E-09 | 5.32E-07 | Cxcl5/Cxcl3/Il6/Il1a/Cx3cl1/Cxcl2/Cntf | 7 | 24.34 | 7/12 | 443/18488 |
| GO:0030593 | neutrophil chemotaxis | 3.38E-09 | 5.32E-07 | Cxcl5/Cxcl3/Cx3cl1/Sell/Cxcl2 | 5 | 76.27 | 5/12 | 101/18488 |
| GO:0097529 | myeloid leukocyte migration | 4.19E-09 | 5.77E-07 | Cxcl5/Cxcl3/Il1a/Cx3cl1/Sell/Cxcl2 | 6 | 38.04 | 6/12 | 243/18488 |
| GO:0071621 | granulocyte chemotaxis | 1.08E-08 | 1.32E-06 | Cxcl5/Cxcl3/Cx3cl1/Sell/Cxcl2 | 5 | 60.66 | 5/12 | 127/18488 |
| GO:0050900 | leukocyte migration | 8.06E-08 | 8.86E-06 | Cxcl5/Cxcl3/Il1a/Cx3cl1/Sell/Cxcl2 | 6 | 23.17 | 6/12 | 399/18488 |
| GO:0070098 | chemokine-mediated signaling pathway | 1.41E-07 | 1.41E-05 | Cxcl5/Cxcl3/Cx3cl1/Cxcl2 | 4 | 79.01 | 4/12 | 78/18488 |
| ID | Description in GO | *P* value | *P.adjust* | Gene ID | Count | Enrich  Score | GeneRatio | BgRatio |
| GO:0042060 | wound healing | 2.07E-07 | 1.71E-05 | Il6/Il1a/Cx3cl1/Cxcl2/Pdgfa/Timp1 | 6 | 19.75 | 6/12 | 468/18488 |
| GO:0030595 | leukocyte chemotaxis | 2.29E-07 | 1.71E-05 | Cxcl5/Cxcl3/Cx3cl1/Sell/Cxcl2 | 5 | 32.92 | 5/12 | 234/18488 |
| GO:1990868 | response to chemokine | 2.41E-07 | 1.71E-05 | Cxcl5/Cxcl3/Cx3cl1/Cxcl2 | 4 | 69.24 | 4/12 | 89/18488 |
| GO:1990869 | cellular response to chemokine | 2.41E-07 | 1.71E-05 | Cxcl5/Cxcl3/Cx3cl1/Cxcl2 | 4 | 69.24 | 4/12 | 89/18488 |
| GO:0010976 | positive regulation of neuron projection development | 2.49E-07 | 1.71E-05 | Cxcl5/Il6/Cx3cl1/Cntf/Ngf | 5 | 32.37 | 5/12 | 238/18488 |
| GO:0070374 | positive regulation of ERK1 and ERK2 cascade | 2.71E-07 | 1.75E-05 | Il6/Il1a/Cx3cl1/Ngf/Pdgfa | 5 | 31.83 | 5/12 | 242/18488 |
| GO:0060326 | cell chemotaxis | 8.94E-07 | 5.46E-05 | Cxcl5/Cxcl3/Cx3cl1/Sell/Cxcl2 | 5 | 25.01 | 5/12 | 308/18488 |
| GO:0071347 | cellular response to interleukin-1 | 1.49E-06 | 8.36E-05 | Il6/Il1a/Cx3cl1/Cxcl2 | 4 | 44.02 | 4/12 | 140/18488 |
| GO:0070372 | regulation of ERK1 and ERK2 cascade | 1.52E-06 | 8.36E-05 | Il6/Il1a/Cx3cl1/Ngf/Pdgfa | 5 | 22.46 | 5/12 | 343/18488 |

Note: Gene Ratio = the gene amount of the corresponding biological process involved by DEPs after CCI / the gene amount of the corresponding biological process in GO database

BgRatio =the rat's gene amount in the GO database is linked to DEPs after CCI/total proteins.

**Table S3.** GO analysed the molecular functions associated with the DEPs between the CCI and control group at 1, 3, 7 days post sciatic nerve injury.

| ID | Description | P value | *P.adjust* | geneID | | Count | Enrich Score | GeneRatio | BgRatio |
| --- | --- | --- | --- | --- | --- | --- | --- | --- | --- |
| **Day1** |  |  |  |  | |  |  |  |  |
| GO:0005125 | cytokine activity | 2.06E-23 | 1.09E-21 | Cxcl1/Cxcl3/Cxcl2/Il1a/Csf2/Cntf/Cx3cl1/Il6/Cxcl5/Vegfa/Ccl2/Ppbp/Timp1 | | 13 | 71.11 | 13/16 | 198/17330 |
| GO:0048018 | receptor ligand activity | 4.36E-23 | 1.13E-21 | Cxcl1/Cxcl3/Cxcl2/Il1a/Ngf/Csf2/Cntf/Cx3cl1/Il6/Cxcl5/Vegfa/Ccl2/Pdgfa/Ppbp/Timp1 | | 15 | 34.35 | 15/16 | 473/17330 |
| GO:0030546 | signaling receptor activator activity | 6.38E-23 | 1.13E-21 | Cxcl1/Cxcl3/Cxcl2/Il1a/Ngf/Csf2/Cntf/Cx3cl1/Il6/Cxcl5/Vegfa/Ccl2/Pdgfa/Ppbp/Timp1 | | 15 | 33.50 | 15/16 | 485/17330 |
| GO:0005126 | cytokine receptor binding | 3.30E-21 | 4.38E-20 | Cxcl1/Cxcl3/Cxcl2/Il1a/Ngf/Csf2/Cntf/Cx3cl1/Il6/Cxcl5/Vegfa/Ccl2/Ppbp | | 13 | 48.55 | 13/16 | 290/17330 |
| GO:0045236 | CXCR chemokine receptor binding | 6.37E-16 | 6.75E-15 | Cxcl1/Cxcl3/Cxcl2/Cx3cl1/Cxcl5/Ppbp | | 6 | 464.20 | 6/16 | 14/17330 |
| GO:0008009 | chemokine activity | 1.25E-15 | 1.10E-14 | Cxcl1/Cxcl3/Cxcl2/Cx3cl1/Cxcl5/Ccl2/Ppbp | | 7 | 204.92 | 7/16 | 37/17330 |
| GO:0042379 | chemokine receptor binding | 1.43E-13 | 1.08E-12 | Cxcl1/Cxcl3/Cxcl2/Cx3cl1/Cxcl5/Ccl2/Ppbp | | 7 | 108.31 | 7/16 | 70/17330 |
| GO:0008083 | growth factor activity | 2.40E-13 | 1.59E-12 | Cxcl1/Ngf/Csf2/Cntf/Il6/Vegfa/Pdgfa/Timp1 | | 8 | 59.76 | 8/16 | 145/17330 |
| GO:0070851 | growth factor receptor binding | 2.84E-09 | 1.67E-08 | Il1a/Csf2/Cntf/Il6/Vegfa/Pdgfa | | 6 | 43.33 | 6/16 | 150/17330 |
| GO:0001664 | G protein-coupled receptor binding | 6.10E-09 | 3.23E-08 | Cxcl1/Cxcl3/Cxcl2/Cx3cl1/Cxcl5/Ccl2/Ppbp | | 7 | 24.07 | 7/16 | 315/17330 |
| GO:0005161 | platelet-derived growth factor receptor binding | 7.23E-05 | 3.48E-04 | Vegfa/Pdgfa | | 2 | 154.73 | 2/16 | 14/17330 |
| GO:0008191 | metalloendopeptidase inhibitor activity | 8.33E-05 | 3.68E-04 | Ngf/Timp1 | | 2 | 144.42 | 2/16 | 15/17330 |
| GO:0042056 | chemoattractant activity | 5.22E-04 | 2.13E-03 | Cx3cl1/Vegfa | | 2 | 58.55 | 2/16 | 37/17330 |
| GO:0048020 | CCR chemokine receptor binding | 9.92E-04 | 3.76E-03 | Cx3cl1/Ccl2 | | 2 | 42.48 | 2/16 | 51/17330 |
| ID | Description | *P* value | *P.adjust* | | geneID | Count | Enrich Score | GeneRatio | BgRatio |
| GO:0019838 | growth factor binding | 7.62E-03 | 2.69E-02 | | Vegfa/Pdgfa | 2 | 15.04 | 2/16 | 144/17330 |
| GO:0002020 | protease binding | 8.57E-03 | 2.84E-02 | | Sell/Timp1 | 2 | 14.16 | 2/16 | 153/17330 |
| GO:0008201 | heparin binding | 9.56E-03 | 2.98E-02 | | Vegfa/Ccl2 | 2 | 13.37 | 2/16 | 162/17330 |
| GO:0048407 | platelet-derived growth factor binding | 1.01E-02 | 2.98E-02 | | Pdgfa | 1 | 98.47 | 1/16 | 11/17330 |
| GO:0005172 | vascular endothelial growth factor receptor binding | 1.19E-02 | 3.16E-02 | | Vegfa | 1 | 83.32 | 1/16 | 13/17330 |
| GO:0033691 | sialic acid binding | 1.19E-02 | 3.16E-02 | | Sell | 1 | 83.32 | 1/16 | 13/17330 |
| **Day3** |  |  |  | |  |  |  |  |  |
| GO:0005125 | cytokine activity | 3.20E-27 | 2.05E-25 | | Cxcl3/Cxcl2/Cxcl1/Il1a/Cxcl5/Vegfa/Ccl2/Il6/Csf2/Il2/Cx3cl1/Il1b/Tnf/Ppbp/Timp1/Cntf | 16 | 63.65 | 16/22 | 198/17330 |
| GO:0048018 | receptor ligand activity | 3.40E-25 | 1.09E-23 | | Cxcl3/Cxcl2/Cxcl1/Il1a/Cxcl5/Vegfa/Ccl2/Pdgfa/Ngf/Il6/Csf2/Il2/Cx3cl1/Il1b/Tnf/Ppbp/Timp1/Cntf | 18 | 29.98 | 18/22 | 473/17330 |
| GO:0030546 | signaling receptor activator activity | 5.36E-25 | 1.14E-23 | | Cxcl3/Cxcl2/Cxcl1/Il1a/Cxcl5/Vegfa/Ccl2/Pdgfa/Ngf/Il6/Csf2/Il2/Cx3cl1/Il1b/Tnf/Ppbp/Timp1/Cntf | 18 | 29.24 | 18/22 | 485/17330 |
| GO:0005126 | cytokine receptor binding | 1.70E-24 | 2.73E-23 | | Cxcl3/Cxcl2/Cxcl1/Il1a/Cxcl5/Vegfa/Ccl2/Ngf/Il6/Csf2/Il2/Cx3cl1/Il1b/Tnf/Ppbp/Cntf | 16 | 43.46 | 16/22 | 290/17330 |
| GO:0045236 | CXCR chemokine receptor binding | 5.92E-15 | 7.58E-14 | | Cxcl3/Cxcl2/Cxcl1/Cxcl5/Cx3cl1/Ppbp | 6 | 337.60 | 6/22 | 14/17330 |
| GO:0008009 | chemokine activity | 1.84E-14 | 1.97E-13 | | Cxcl3/Cxcl2/Cxcl1/Cxcl5/Ccl2/Cx3cl1/Ppbp | 7 | 149.03 | 7/22 | 37/17330 |
| GO:0008083 | growth factor activity | 7.09E-14 | 6.48E-13 | | Cxcl1/Vegfa/Pdgfa/Ngf/Il6/Csf2/Il2/Timp1/Cntf | 9 | 48.89 | 9/22 | 145/17330 |
| GO:0042379 | chemokine receptor binding | 2.09E-12 | 1.68E-11 | | Cxcl3/Cxcl2/Cxcl1/Cxcl5/Ccl2/Cx3cl1/Ppbp | 7 | 78.77 | 7/22 | 70/17330 |
| GO:0070851 | growth factor receptor binding | 7.53E-12 | 5.36E-11 | | Il1a/Vegfa/Pdgfa/Il6/Csf2/Il2/Il1b/Cntf | 8 | 42.01 | 8/22 | 150/17330 |
| ID | Description | *P* value | *P.adjust* | | geneID | Count | Enrich Score | GeneRatio | BgRatio |
| GO:0001664 | G protein-coupled receptor binding | 2.79E-09 | 1.79E-08 | | Cxcl3/Cxcl2/Cxcl1/Cxcl5/Ccl2/Il2/Cx3cl1/Ppbp | 8 | 20.01 | 8/22 | 315/17330 |
| GO:0005161 | platelet-derived growth factor receptor binding | 1.39E-04 | 8.07E-04 | | Vegfa/Pdgfa | 2 | 112.53 | 2/22 | 14/17330 |
| GO:0008191 | metalloendopeptidase inhibitor activity | 1.60E-04 | 8.53E-04 | | Ngf/Timp1 | 2 | 105.03 | 2/22 | 15/17330 |
| GO:0005149 | interleukin-1 receptor binding | 1.83E-04 | 8.99E-04 | | Il1a/Il1b | 2 | 98.47 | 2/22 | 16/17330 |
| GO:0050839 | cell adhesion molecule binding | 4.26E-04 | 1.95E-03 | | Sell/Cx3cl1/Icam1/Il1b | 4 | 10.98 | 4/22 | 287/17330 |
| GO:0051861 | glycolipid binding | 6.12E-04 | 2.61E-03 | | Sell/Il2 | 2 | 54.33 | 2/22 | 29/17330 |
| GO:0005178 | integrin binding | 8.02E-04 | 3.21E-03 | | Cx3cl1/Icam1/Il1b | 3 | 16.19 | 3/22 | 146/17330 |
| GO:0002020 | protease binding | 9.19E-04 | 3.46E-03 | | Sell/Tnf/Timp1 | 3 | 15.45 | 3/22 | 153/17330 |
| GO:0042056 | chemoattractant activity | 9.97E-04 | 3.55E-03 | | Vegfa/Cx3cl1 | 2 | 42.58 | 2/22 | 37/17330 |
| GO:0032813 | tumor necrosis factor receptor superfamily binding | 1.61E-03 | 5.41E-03 | | Ngf/Tnf | 2 | 33.52 | 2/22 | 47/17330 |
| GO:0048020 | CCR chemokine receptor binding | 1.89E-03 | 6.04E-03 | | Ccl2/Cx3cl1 | 2 | 30.89 | 2/22 | 51/17330 |
| **Day7** |  |  |  | |  |  |  |  |  |
| GO:0048018 | receptor ligand activity | 1.31E-14 | 3.97E-13 | | Cxcl5/Cxcl3/Il6/Il1a/Cx3cl1/Cxcl2/Cntf/Ngf/Pdgfa/Timp1 | 10 | 30.53 | 10/12 | 473/17330 |
| GO:0030546 | signaling receptor activator activity | 1.69E-14 | 3.97E-13 | | Cxcl5/Cxcl3/Il6/Il1a/Cx3cl1/Cxcl2/Cntf/Ngf/Pdgfa/Timp1 | 10 | 29.78 | 10/12 | 485/17330 |
| GO:0005125 | cytokine activity | 1.20E-13 | 1.88E-12 | | Cxcl5/Cxcl3/Il6/Il1a/Cx3cl1/Cxcl2/Cntf/Timp1 | 8 | 58.35 | 8/12 | 198/17330 |
| GO:0005126 | cytokine receptor binding | 2.61E-12 | 3.07E-11 | | Cxcl5/Cxcl3/Il6/Il1a/Cx3cl1/Cxcl2/Cntf/Ngf | 8 | 39.84 | 8/12 | 290/17330 |

| ID | Description | *P* value | *P.adjust* | geneID | Count | Enrich Score | GeneRatio | BgRatio |
| --- | --- | --- | --- | --- | --- | --- | --- | --- |
| GO:0045236 | CXCR chemokine receptor binding | 1.31E-10 | 1.24E-09 | Cxcl5/Cxcl3/Cx3cl1/Cxcl2 | 4 | 412.62 | 4/12 | 14/17330 |
| GO:0008009 | chemokine activity | 8.60E-09 | 6.73E-08 | Cxcl5/Cxcl3/Cx3cl1/Cxcl2 | 4 | 156.13 | 4/12 | 37/17330 |
| GO:0008083 | growth factor activity | 2.89E-08 | 1.94E-07 | Il6/Cntf/Ngf/Pdgfa/Timp1 | 5 | 49.80 | 5/12 | 145/17330 |
| GO:0042379 | chemokine receptor binding | 1.18E-07 | 6.93E-07 | Cxcl5/Cxcl3/Cx3cl1/Cxcl2 | 4 | 82.52 | 4/12 | 70/17330 |
| GO:0070851 | growth factor receptor binding | 2.53E-06 | 1.32E-05 | Il6/Il1a/Cntf/Pdgfa | 4 | 38.51 | 4/12 | 150/17330 |
| GO:0008191 | metalloendopeptidase inhibitor activity | 4.59E-05 | 2.02E-04 | Ngf/Timp1 | 2 | 192.56 | 2/12 | 15/17330 |
| GO:0001664 | G protein-coupled receptor binding | 4.72E-05 | 2.02E-04 | Cxcl5/Cxcl3/Cx3cl1/Cxcl2 | 4 | 18.34 | 4/12 | 315/17330 |
| GO:0002020 | protease binding | 4.82E-03 | 1.89E-02 | Sell/Timp1 | 2 | 18.88 | 2/12 | 153/17330 |
| GO:0048407 | platelet-derived growth factor binding | 7.59E-03 | 2.50E-02 | Pdgfa | 1 | 131.29 | 1/12 | 11/17330 |
| GO:0004866 | endopeptidase inhibitor activity | 7.80E-03 | 2.50E-02 | Ngf/Timp1 | 2 | 14.74 | 2/12 | 196/17330 |
| GO:0030414 | peptidase inhibitor activity | 8.34E-03 | 2.50E-02 | Ngf/Timp1 | 2 | 14.23 | 2/12 | 203/17330 |
| GO:0033691 | sialic acid binding | 8.97E-03 | 2.50E-02 | Sell | 1 | 111.09 | 1/12 | 13/17330 |
| GO:0061135 | endopeptidase regulator activity | 9.23E-03 | 2.50E-02 | Ngf/Timp1 | 2 | 13.50 | 2/12 | 214/17330 |
| GO:0005161 | platelet-derived growth factor receptor binding | 9.65E-03 | 2.50E-02 | Pdgfa | 1 | 103.15 | 1/12 | 14/17330 |
| GO:0005149 | interleukin-1 receptor binding | 1.10E-02 | 2.50E-02 | Il1a | 1 | 90.26 | 1/12 | 16/17330 |
| GO:0005165 | neurotrophin receptor binding | 1.10E-02 | 2.50E-02 | Ngf | 1 | 90.26 | 1/12 | 16/17330 |

Note: Gene Ratio = the gene amount of the corresponding molecular functions involved by DEPs after CCI / the gene amount of the corresponding molecular functions in GO database

BgRatio =the rat's gene amount in the GO database is linked to DEPs after CCI/total proteins.

**Table S4.** GO analysed the cellular components associated with the DEPs between the CCI and control group at 3 days post sciatic nerve injury.

| ID | Description | *P* value | *P.adjust* | geneID | Count | Enrich Score | GeneRatio | BgRatio |
| --- | --- | --- | --- | --- | --- | --- | --- | --- |
| **Day3** |  |  |  |  |  |  |  |  |
| GO:0009897 | external side of plasma membrane | 1.00E-05 | 3.61E-04 | Sell/Il6/Icam1/Tnf/Cd86/Prlr | 6 | 11.35 | 6/22 | 450/18728 |
| GO:0005604 | basement membrane | 7.19E-03 | 4.95E-02 | Vegfa/Timp1 | 2 | 15.62 | 2/22 | 109/18728 |
| GO:0043235 | receptor complex | 1.10E-02 | 4.95E-02 | Il6/Csf2/Prlr | 3 | 6.38 | 3/22 | 400/18728 |
| GO:0070062 | extracellular exosome | 1.12E-02 | 4.95E-02 | Icam1/Cd86 | 2 | 12.43 | 2/22 | 137/18728 |
| GO:0031904 | endosome lumen | 1.17E-02 | 4.95E-02 | Ngf | 1 | 85.13 | 1/22 | 10/18728 |
| GO:0005796 | Golgi lumen | 1.28E-02 | 4.95E-02 | Ngf | 1 | 77.39 | 1/22 | 11/18728 |
| GO:0030141 | secretory granule | 1.32E-02 | 4.95E-02 | Vegfa/Il1b/Ppbp | 3 | 5.97 | 3/22 | 428/18728 |
| GO:1903561 | extracellular vesicle | 1.34E-02 | 4.95E-02 | Icam1/Cd86 | 2 | 11.28 | 2/22 | 151/18728 |
| GO:0043230 | extracellular organelle | 1.38E-02 | 4.95E-02 | Icam1/Cd86 | 2 | 11.13 | 2/22 | 153/18728 |
| GO:0065010 | extracellular membrane-bounded organelle | 1.38E-02 | 4.95E-02 | Icam1/Cd86 | 2 | 11.13 | 2/22 | 153/18728 |

Note: Gene Ratio = the gene amount of the corresponding cellular components involved by DEPs after CCI / the gene amount of the corresponding cellular components in GO database

BgRatio =the rat's gene amount in the GO database is linked to DEPs after CCI/total proteins.
